# Integrating Photoactive Ligands into Crystalline Ultrathin 2D Metal–Organic Framework Nanosheets for Efficient Photoinduced Energy Transfer

**DOI:** 10.1021/jacs.3c10917

**Published:** 2024-01-03

**Authors:** Hengyu Lin, Yihao Yang, Brian G. Diamond, Tian-Hao Yan, Vladimir I. Bakhmutov, Kelechi W. Festus, Peiyu Cai, Zhifeng Xiao, Mingwan Leng, Ibukun Afolabi, Gregory S. Day, Lei Fang, Christopher H. Hendon, Hong-Cai Zhou

**Affiliations:** † Department of Chemistry, Texas A&M University, College Station, Texas 77843, United States; ‡ Department of Chemistry, University of Oregon, Eugene, Oregon 97403, United States

## Abstract

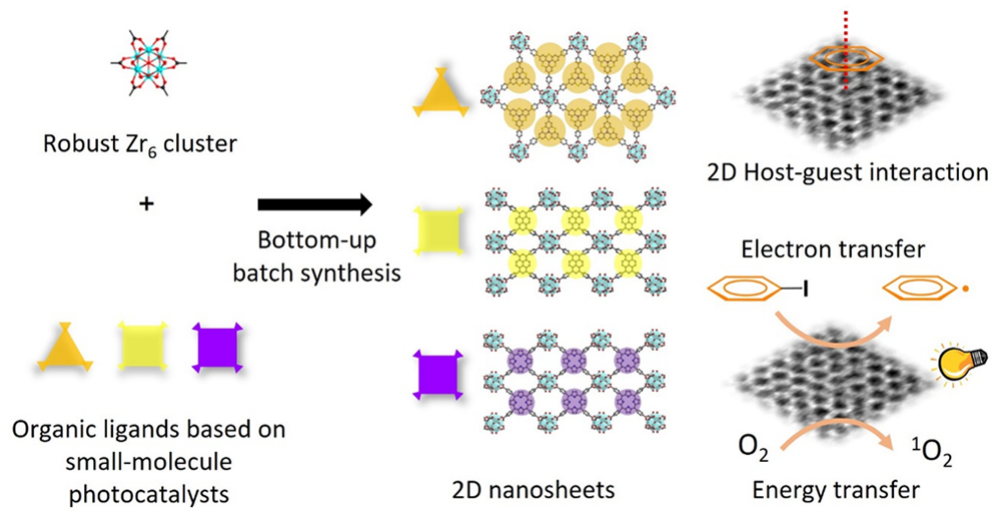

3D metal–organic frameworks (MOFs) have gained attention as heterogeneous photocatalysts due to their porosity and unique host–guest interactions. Despite their potential, MOFs face challenges, such as inefficient mass transport and limited light penetration in photoinduced energy transfer processes. Recent advancements in organic photocatalysis have uncovered a variety of photoactive cores, while their heterogenization remains an underexplored area with great potential to build MOFs. This gap is bridged by incorporating photoactive cores into 2D MOF nanosheets, a process that merges the realms of small-molecule photochemistry and MOF chemistry. This approach results in recyclable heterogeneous photocatalysts that exhibit an improved mass transfer efficiency. This research demonstrates a bottom-up synthetic method for embedding photoactive cores into 2D MOF nanosheets, successfully producing variants such as PCN-641-NS, PCN-643-NS, and PCN-644-NS. The synthetic conditions were systematically studied to optimize the crystallinity and morphology of these 2D MOF nanosheets. Enhanced host–guest interactions in these 2D structures were confirmed through various techniques, particularly solid-state NMR studies. Additionally, the efficiency of photoinduced energy transfer in these nanosheets was evidenced through photoborylation reactions and the generation of reactive oxygen species (ROS).

## Introduction

2D nanomaterials are an emerging field in material science.^1^ Since the discovery of graphene, transition metal dichalcogenides (TMDs),^2−4^ graphitic carbon nitride (g-C_3_N_4_), hexagonal boron nitride (h-BN),^5−7^ layered double hydroxides (LDHs),^8^ black phosphorus (BP),^9^ oxides,^10^ and Mxenes^11,12^ have intrigued researchers by their unique properties. The atomic-level thickness of a one-dimensional material adds unique characteristics to these materials. Their high exposed surface area, optical transparency, and excellent electric and thermal conductivity make them competitive candidate materials for gas separation, energy conversion, catalysis, and sensing. Retrospectively, the formation of 2D nanomaterials has laid an emphasis on inorganic/atomic level fabrication such as the precise disposition of metal ions and counterions, carbon, and other atoms, while the utilization of a coordination bond and the incorporation of organic molecules in 2D nanomaterials’ formation by the coordination bond are less explored.

Organic photocatalysts have been enormously developed and yielded a fruitful area with hundreds of reactions achievable via light excitation.^13^ Introducing photoactive organic molecules into 2D materials can largely maintain their photophysics and simultaneously enhance the robustness and viability of the active centers. Therefore, it is important to explore the introduction of organic photocatalysts into robust 2D materials for more tailorable and viable heterogeneous photocatalysts.

2D metal–organic frameworks (MOFs), with their intrinsic porosity, are one of the fastest growing materials in catalysis.^14,15^ One strength of MOFs is their ligand-based chemical property design. Also, the introduction of various metal clusters enriches the topological design. Both factors result in high structural tunability of MOFs. Via the rational selection of metal clusters, ligands of various connectivities can be implemented in MOFs. This allows for control over not only the porosity and pore environment but also the photophysics and activity.^16^ Additionally, their crystallinity allows them to be studied with multiple crystallographic methodologies (single-crystal, powder X-ray diffraction, etc.), providing explicit structural information. 2D MOF nanosheets have drawn researchers’ attention due to their highly exposed surface area and atomic-level thickness, which allow for efficient diffusion of substrates and products and a different type of host–guest interaction by surface-anchoring.^17,18^ Under controlled conditions, MOF nanosheets can be obtained from top-down and bottom-up synthetic routes.^19,20^ In top-down synthesis, bulk 3D MOFs are initially grown, followed by the application of mechanical forces (stirring, grinding, sonication, etc.) on the bulk materials to exfoliate ultrathin layers of 2D MOFs. This requires the bulk MOFs to have relatively lower interlayer interactions. It is worth noting that other than mechanical exfoliation, “chemical exfoliation” has been developed to control the exfoliation process where a pillar ligand with a labile bond can be introduced to assist the formation of 3D layered MOF crystals and afterward can be cleaved by chemical treatment to precisely exfoliate single layers of 2D MOFs.^21,22^ Instead, bottom-up synthesis skips the formation of 3D bulk MOFs by directly assembling the metal clusters with the organic linkers to build 2D MOFs de novo. The synthetic conditions are more intricate to control, and multiphase products can often be yielded.^23,24^

The fascinating features of 2D MOF nanosheets have led to extensive studies of heterogeneous catalysis. Heterogeneous catalytic activity can be introduced into 2D MOFs via various design approaches: 1) catalytic functionalities (organic and metal catalysts) can be anchored onto 2D nanosheets through coordinative or covalent bonding,^25,26^ 2) metal clusters can participate in the catalytic cycle directly,^27^ or 3) the MOF nanosheets are produced from intrinsically active ligands.^23,28^ Herein, this work explores path 3) with the intention to build a nonunique synthetic strategy to incorporate photoactive cores into 2D MOFs for more efficient heterogeneous photocatalysis. In this scope, ligands with various reaction specialties can be introduced to enable ^1^O_2_ generation, CO_2_ reduction, and other organic transformations through electron and energy transfer.

Phenothiazine (PTH) is a sulfur- and nitrogen-based photoresponsive heterocycle. This small molecule shows high reductive potential upon light excitation (*E*_*s*1_ = −2.1 V vs saturated calomel electrode).^29^ The high redox capability makes it a good candidate for photoinduced electron transfer catalysis. Multiple reports have shown its excellent performance in hydrodehalogenation of arylhalides,^30^ atom-transfer radical polymerization (ATRP),^31^ and interrupted Pummerer-activated formal C–H/C–H coupling of arenes.^32^ However, organic photocatalysts suffer from photobleaching and short catalyst lifetimes. Crafted into an MOF, PTH gains extra stability and robustness in the framework. Additionally, the fabrication of PTH into a MOF turns it from a homogeneous system to a heterogeneous system, enabling facile catalyst recycling. This work showcases the benefits of 2D MOFs as platforms for photocatalyst heterogenization.

As a catalyst-bearing platform, 2D MOF nanosheets can be more competitive than traditional 3D MOFs. Limited by the relatively large size of 3D bulk MOFs, substrate diffusion is typically limited to the surface crystal cells, leaving the inner pores and catalytic sites unused. Also, the bulk structure of 3D MOFs limits light penetration. 2D MOF nanosheets solve the diffusion and light penetration limitation problems, while maintaining MOFs’ tunability in terms of cluster, ligand, and postsynthetic modification (PSM) choices.

**Figure 1:**
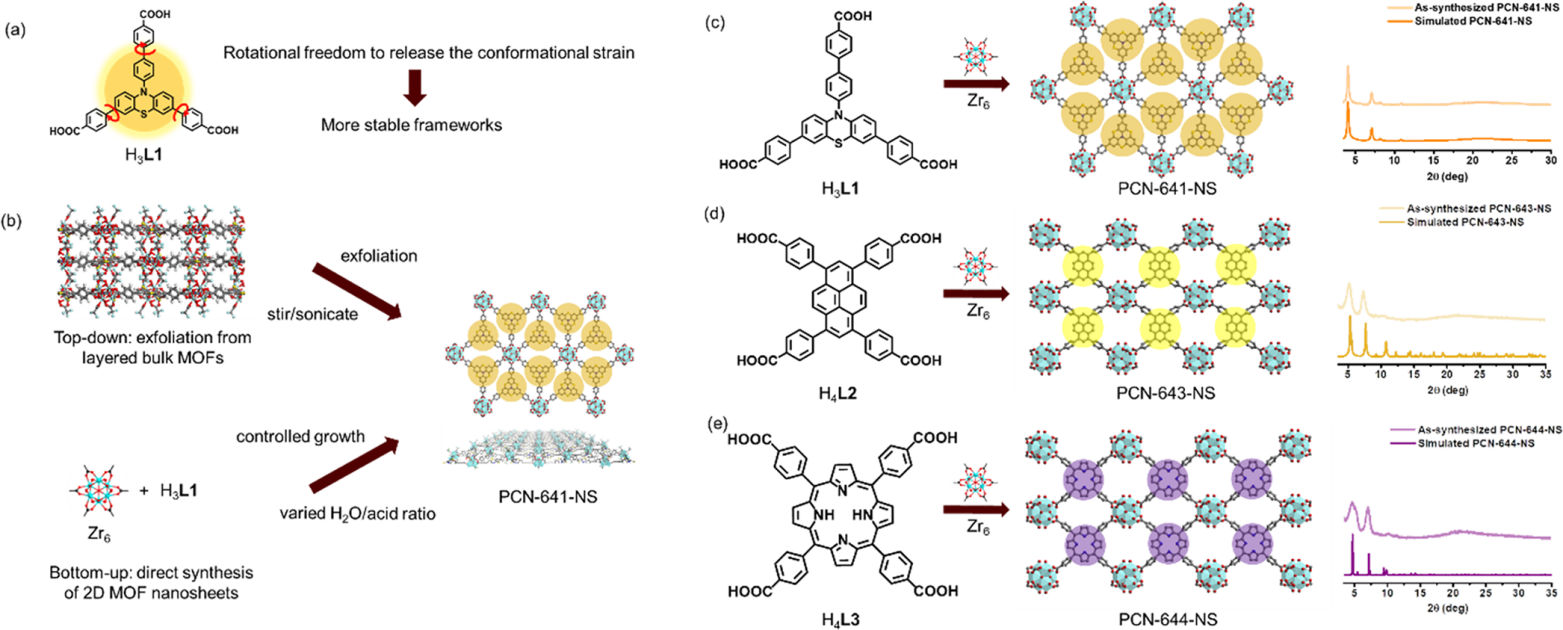
Schematic illustration of ligand choice and top-down, bottom-up syntheses of 2D MOF nanosheets. (a) The chemical structure of ligand H_3_**L1**, the phenyl groups between the PTH core and carboxylic groups introducing rotational freedom. (b) (Left) top-down synthesis of PCN-641-NS, exfoliation from bulk PCN-641. (Right) bottom-up synthesis of PCN-641-NS, assembly of Zr_6_ clusters and H_3_**L1.** Bottom-up synthesis of (c) PCN-641-NS, (d) PCN-643-NS, and (e) PCN-644-NS and respective powder X-ray match with simulated structures.

In this work, a photosensitive tricarboxylate ligand, H_3_**L1**, is developed from PTH with a 4-carboxyphenyl substitution on each direction (Figure 1a). The phenyl groups add rotational freedom to relieve the conformational strain and form stable 2D MOFs. Top-down and bottom-up syntheses were demonstrated, and the 2D MOF nanosheet, PCN-641-NS, was subsequently obtained (Figure 1b-c). Among them, the bottom-up approach showed excellent reaction yield and crystallinity after condition screening. The nanosheets showed excellent performance as photocatalysts in the photoborylation of aryl halides. The same fabrication strategy was applied to other photoactive cores, pyrene and porphyrin, to obtain PCN-643-NS and PCN-644-NS and demonstrate the nonunique bottom-up synthesis of 2D MOF nanosheets (Figure 1d-e and Figure S1b-c). Ultrathin layers of 2D MOFs were obtained and characterized by electron microscopy and atomic force microscopy. Moreover, the heterogeneous catalyst-substrate interaction was explored by solid-state NMR and photoluminescence studies. In this study, pillar-layered PCN-642 was obtained as the 3D analogous comparison of PCN-641-NS. The catalyzed photoborylation and reactive oxygen species (ROS) generation were conducted to prove the elevated catalytic activities of nanosheets compared to bulk MOFs.

## Results and Discussion

### Bottom-up Synthesis of PCN-641-Nanosheets (NS) and Characterizations

**Figure 2:**
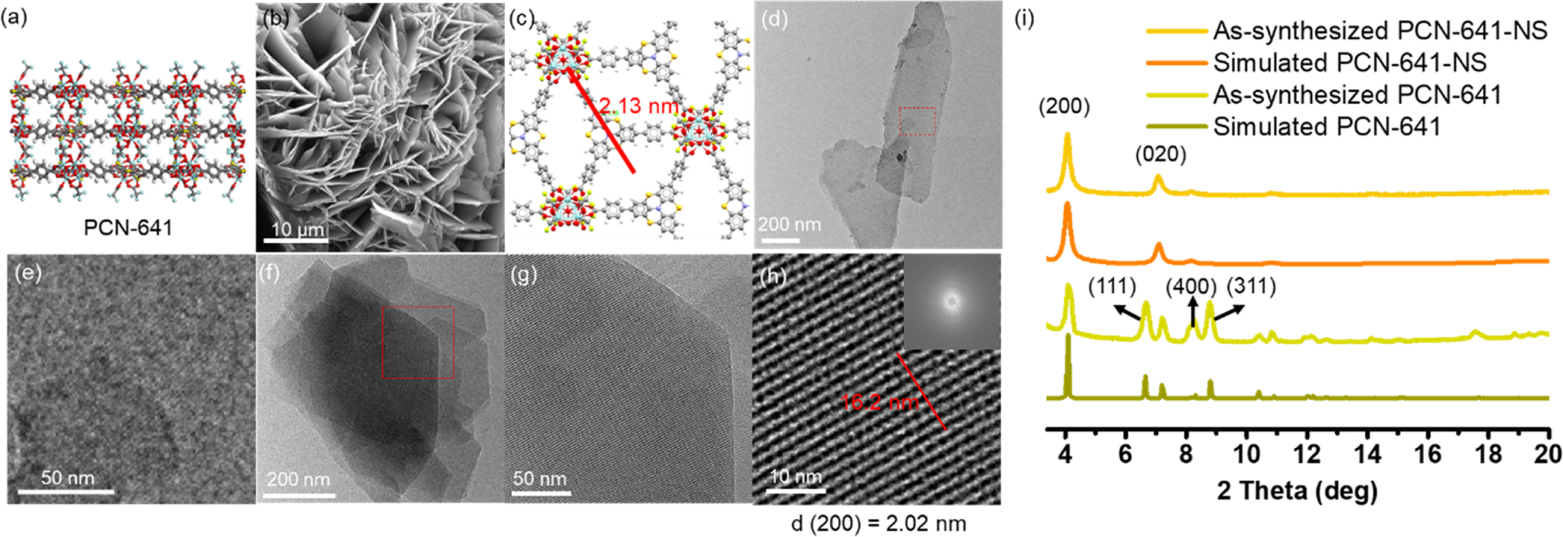
(a) Simulated crystal structure of PCN-641. (b) SEM image of the clustered PCN-641 flakes, scale bar: 10 μm. (c) Simulated structure of PCN-641-NS with d(200) measured to be 2.13 nm. (d) TEM image of top-down exfoliated PCN-641-NS, scale bar: 200 nm. (e) Zoom-in TEM image of (d), scale bar: 50 nm. (f) TEM image of bottom-up synthesized PCN-641-NS, scale bar: 200 nm. (g) Zoom-in TEM image of (f), scale bar: 50 nm. (h) HR-TEM image of bottom-up synthesized PCN-641-NS with d(200) spacing measured to be 2.02 nm, scale bar: 10 nm. (i) PXRD patterns of simulated and as-synthesized PCN-641 and PCN-641-NS. The diffractions are indexed in Jade with Miller indices calculated and labeled in the graph.

A Zr_6_ cluster was selected to build 2D MOFs due to its robustness and minor effects on the photoactivity of ligands. Length-optimized ligands with tri/tetratopic connectivity have the tendency to form layered MOF frameworks. A bulk 3D MOF with a layered structure, PCN-641, was obtained by ligand H_3_**L1** and the Zr_6_ cluster, its morphology was shown by SEM, and its structure was confirmed by a powder X-ray diffraction pattern matching the simulated pattern (Figure 2a-b,i). Top-down and bottom-up syntheses of PCN-641-NS were demonstrated (Supporting Information Section 5) and compared. After applying mechanical forces to bulk PCN-641 dispersed in ethanol, we exfoliated thin layers of MOFs and observed them under TEM (Figure 2d-e). However, the exfoliation yield is too low to be calculated, only providing nanosheet solutions for TEM. The bottom-up route can yield more regular sizes of PCN-641-NS as shown by TEM (Figure 2f-g). Using high-resolution TEM (HR-TEM, Figure 2h), the crystal lattice d(200) spacing can be measured as 2.02 nm, which matches the simulated PCN-641-NS structure (2.13 nm, as shown in Figure 2c). Indexed by Jade, the PXRD results of PCN-641-NS show two major peaks [(200) and (020)] without observable stacking, while PCN-641’s results include (200), (111), (020), (400), and (311). This indicates the stacking of layers in PCN-641. Instead of hexagonal pore structures, the TEM image shows stripes along one direction. This is due to the AB-stacking of nanosheets, which allows us to merely observe one type of lattice distance. Structure simulation shows that AB-packed PCN-641-NS’s PXRD after Rietveld refinement matches the as-synthesized PXRD results with an Rwp of 6.74%, which is in accordance with the AB-packing observed by HR-TEM (Figure S11). PCN-641-NS’s structure can be found in a cif file. It is worth mentioning that the bottom-up route provides reliable repeatability and decent yield (35%) for repeatable batch synthesis.

It is important to understand how varied conditions contribute to the morphological tuning of the nanosheet growth. Water and monotopic carboxylic acids are added in the synthetic solutions to control the framework formation kinetics. Compared to 3D MOF synthesis, relatively higher water concentration can limit the growth to 2D. Formic acid (FA), acetic acid (AA), trifluoroacetic acid (TFA), propanoic acid (PA), caproic acid (CA), and benzoic acid (BA) were studied, and the resultant morphology was examined by SEM and TEM (Table S1 and Figures S3–S8, S10). FA and TFA yielded monolayers with good crystallinity as revealed by the SEM (Figures S3–S4) and TEM images (Figure S10a-b) and PXRD patterns (Figures S12–S13). With the addition of water, a decrease in the nanosheet size is observed in all SEM images with different acids. In the AA, CA, PA, and BA series, the synthetic mixture yields tiny beads or cubes with low crystallinity without the addition of water (Figures S5–S8 and Figures S14–S17). To our expectation, when water was added, a transition from a 3D to 2D morphology is observed under SEM, possibly due to the increased amount of Zr_6_ clusters present in the solution, whose formation is driven by water reacting with ZrCl_4_. Synthesized with AA, CA, PA, and BA, the 2D nanosheets have a considerable amount of “wet-paper”-like morphology as shown by TEM (Figure S10c-f) with lower crystallinity (Figures S14–S17), which can be the result of hydrophobic interactions among alkyl/phenyl groups of the carboxylic acids. FA-modulated PCN-641-NS shows the best crystallinity in PXRD with clear lattice arrays under TEM (Figures S9a, S12). TFA-modulated PCN-641-NS displays a lower crystallinity, yet lattice arrays can still be observed (Figures S9b, S13). Other modulator acids show thin layers with low crystallinity as evidenced by the rise of amorphous peaks between 15 and 30° in PXRD. Thus, the FA-modulated recipe was taken as the method for batch synthesis.

**Figure 3:**
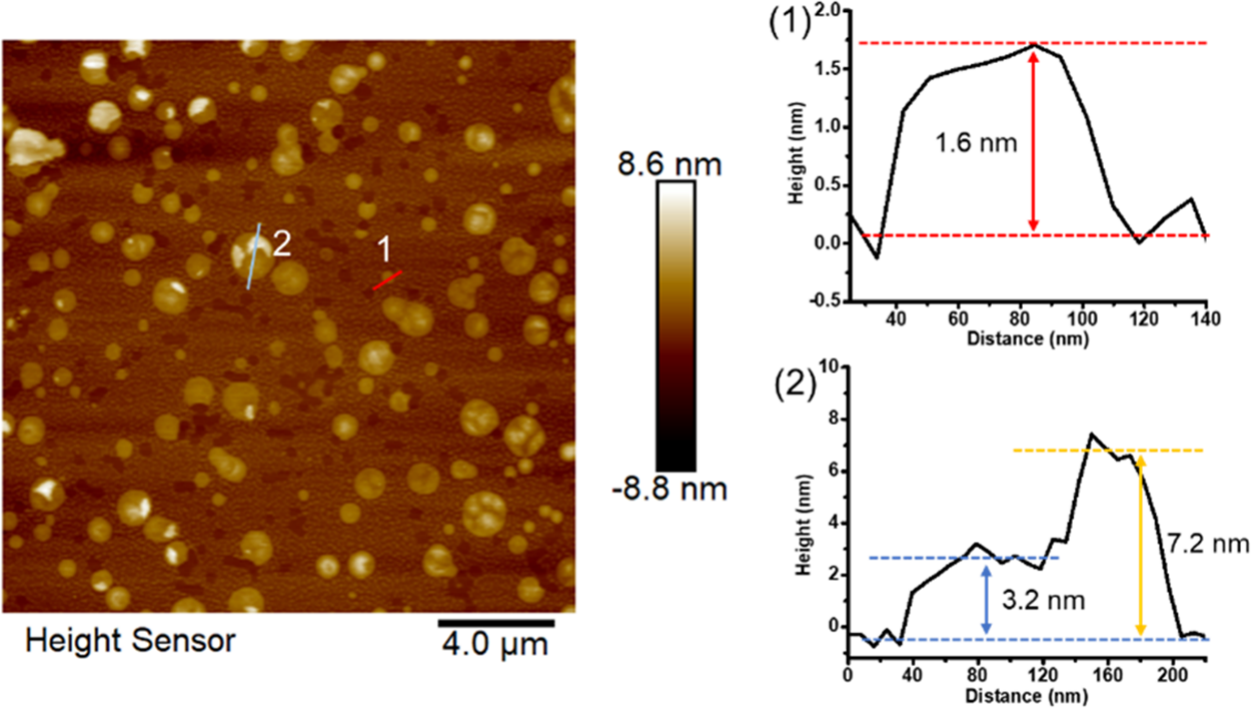
AFM images of PCN-641-NS. Two linear regions of interest (ROI) are picked for height measurement (1 and 2 corresponding to the height profiles).

The thickness measurement confirmed the thin feature of the 2D MOF nanosheets. 2D MOF nanosheets are defined as thickness < 10 nm. As shown in Figure 3, atomic force microscopy (AFM) confirmed the thickness of the as-synthesized PCN-641-NS to be 1.6 nm, which is the monolayer height of this structure. Also, thicker layers have been observed (3–7 nm), indicating that there are also multiple-layered nanosheets present (2–4 layers).

### Other Photoactive Catalysts PCN-643-NS and PCN-644-NS

The same synthetic strategy can be applied to other photoactive ligands, such as pyrene and porphyrin. The pyrene and porphyrin cores, through multistep and single-step syntheses, can be modified into tetra-topic ligands H_4_**L2** and H_4_**L3**. Assembled with Zr_6_ clusters, PCN-643-NS and PCN-644-NS were yielded, whose structures were confirmed by PXRD matching with simulated structures (Figure 1d-e). The crystallinity is lower than PCN-641-NS from the broadened diffraction peaks largely as a result of elevated interlayer interactions from larger aromatic systems. The morphology can be confirmed by SEM images (Figure S9), and the thin feature can be measured by AFM (Figures S19–S20). Simulated structures for PXRD matching can be found in the cif files. This demonstrates that the strategy can be applied to other photoactive ligands.

### Partially Oxidized PCN-641-NSO for Reduced Packing

**Figure 4:**
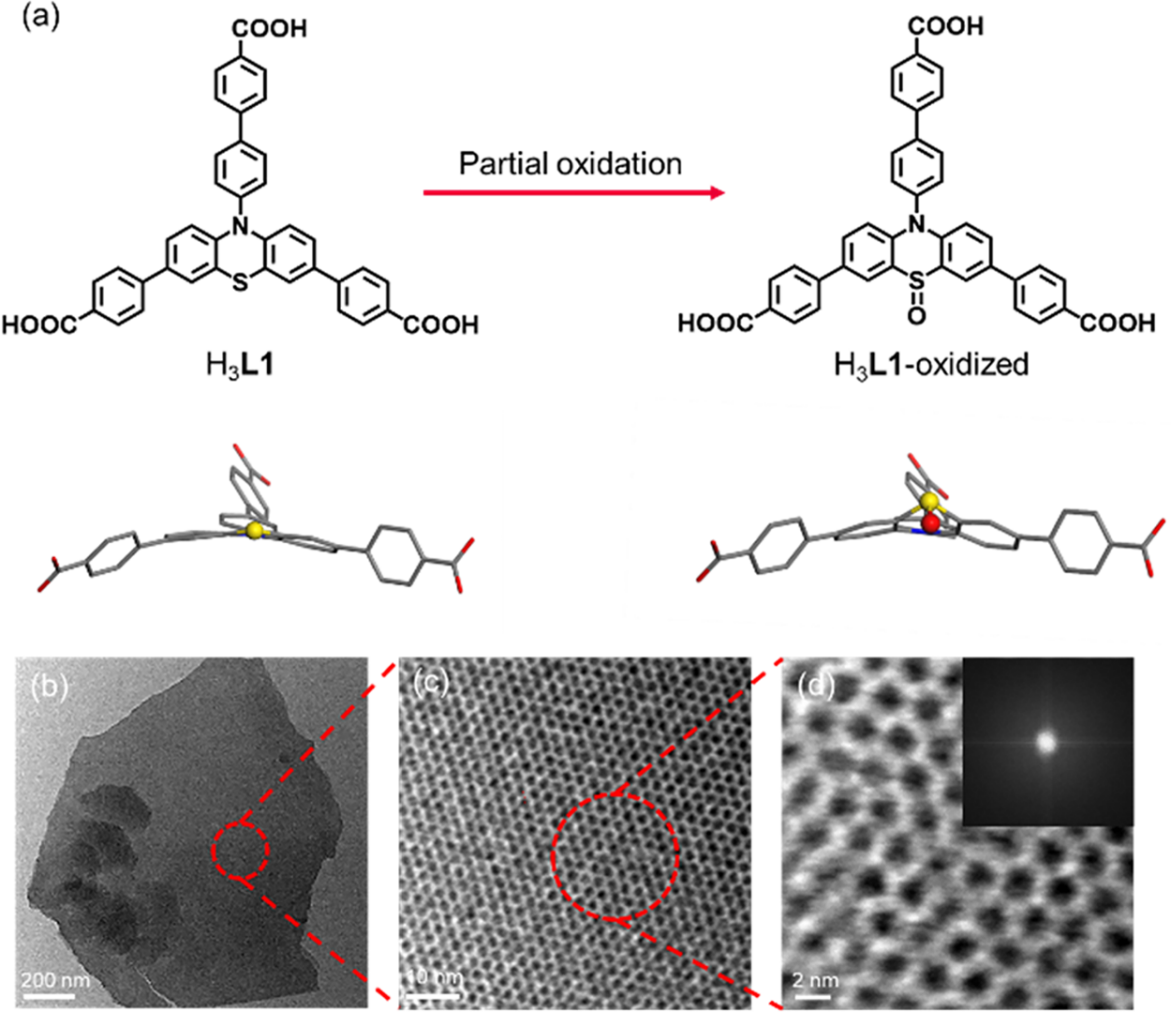
(a) Partial oxidation of H_3_**L1** into H_3_**L1**-oxidized and sulfur turned into sulfoxide. (b) TEM image of PCN-641-NSO, scale bar: 200 nm. (c) Zoom-in area of PCN-641-NSO, scale bar: 10 nm. (d) HR-TEM zoom-in area of PCN-641-NSO, scale bar: 2 nm.

The S atom in the phenothiazine core of H_3_**L1** is susceptible to oxidation under synthetic temperature and aerobic conditions. Upon structural simulation using H_3_**L1**-oxidized, an out-of-plane distortion of phenothiazine can be caused by the addition of oxygen (Figure 4a). This can lead to a reduction of stacking and result in the observed well-resolved porous structure. This transformation might rigidify the framework structure by making the conformation of phenothiazine fixed. The partially oxidized nanosheet is named PCN-641-NSO. A well-resolved hexagonal porous structure was observed under HR-TEM, and the pore size matches the simulated PCN-641-NS structure (Figure 4b-d).

### Pore Volume and Surface Area

**Figure 5:**
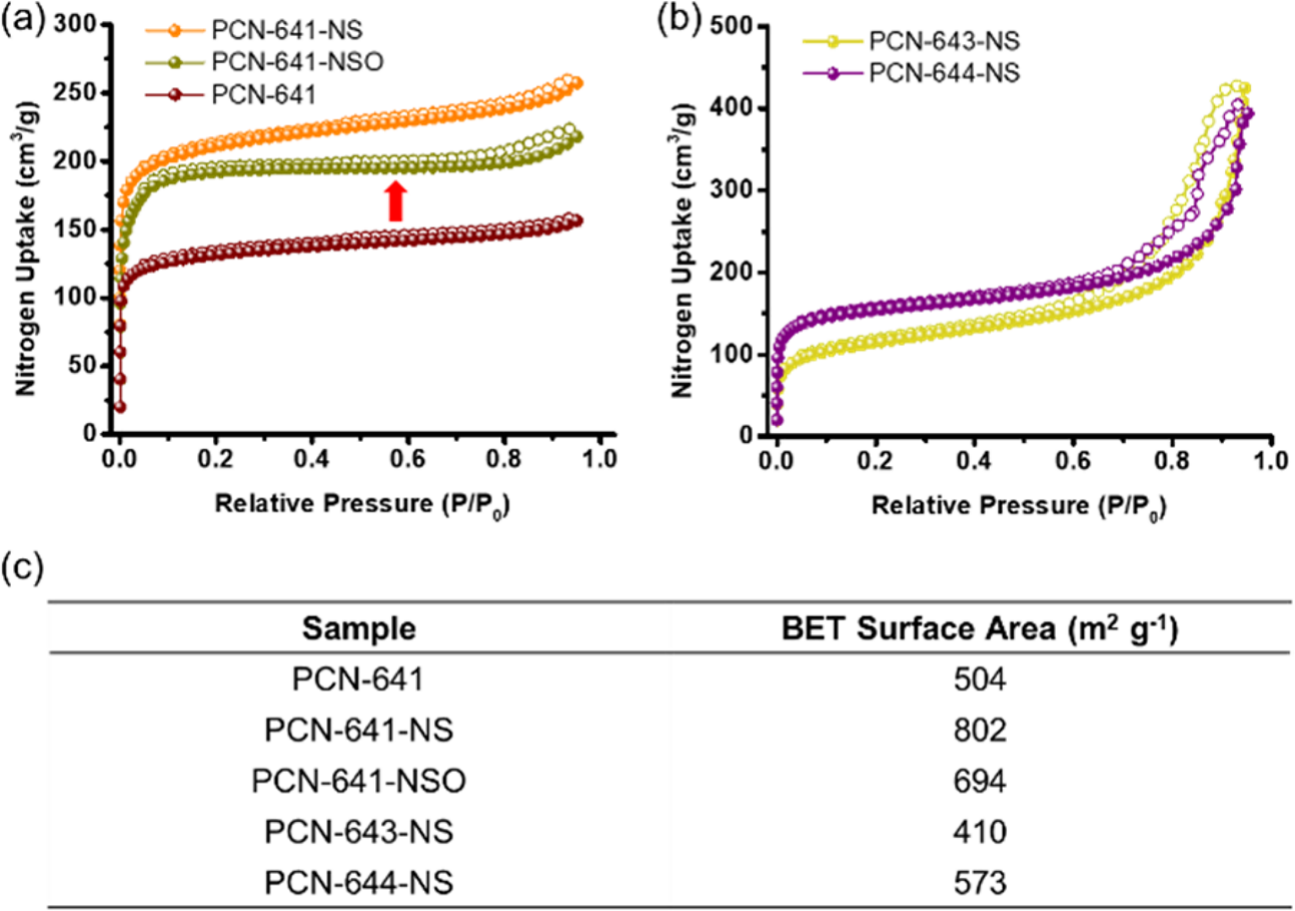
(a) N_2_ sorption isotherms of PCN-641, PCN-641-NS, and PCN-641-NSO. (b) N_2_ sorption isotherms of PCN-643-NS and PCN-644-NS. (c) BET surface area of 3D and 2D MOFs.

The surface area of the MOFs can represent the number of active sites exposed. Brunauer–Emmett–Teller (BET) N_2_ sorption isotherm is a widely acknowledged measurement to characterize the pore volume and surface area. However, it should be noted that in catalytic reactions the substrate molecules with varied sizes might not have the same permeability to access the active sites. The fact that the “probe molecule”, N_2_, readily accesses almost all the micropores leads to the overestimation of surface area open for catalysis. Therefore, even provided that 3D MOFs might show a fascinating BET surface area, they might not provide so many accessible photoactive sites as 2D MOFs, while the dispersion of 2D MOF nanosheets in solution exposes the surface area and enables all active sites to be accessible. The N_2_ isotherms were collected after pore activation and summarized in Figure 5a-c. Interestingly, due to the relatively large pore size to fit the width ligands, interpenetration of layers exists in bulk PCN-641, which leads to the clogging of pores. Thus, a rise in BET surface area was observed from PCN-641 to PCN-641-NS and PCN-641-NSO (from 504 m^2^ g^–1^ to 802 m^2^ g^–1^ and 694 m^2^ g^–1^). The higher surface area indicates more accessible active sites.

The DFT calculated micropore size distribution matches the simulated framework structures of PCN-641-NS, PCN-643-NS, and PCN-644-NS (Figure S21). Compared to bulk PCN-641 (Figure S21a), PCN-641-NS’s pore size distribution is more defined (Figure S21b). A peak indicating a one-ligand defect is observed in PCN-641-NS and PCN-641-NSO around 15–19 Å. This indicates the possibility of anchoring a size-fitting catalyst at the defect site for tandem catalytic platforms. The BJH desorption calculated mesoporosity distribution shows that PCN-641-NS has negligible distribution in the mesoporous range (Figure S22b), while PCN-643-NS and PCN-644-NS present an extent of mesoporosity (Figure S22c-d). Given that PCN-641-NS is crystalline, the pore size distribution is more uniform in the microporous range. Since PCN-643-NS and PCN-644-NS have stronger interlayer interactions, stacking-caused mesopores rise in the solid materials. PCN-643-NS, based on pyrenes that can be “sticky” to each other, shows a wide distribution and high pore volume created by stacking.

### Host–Guest Interaction in 3D and 2D Frameworks

**Figure 6:**
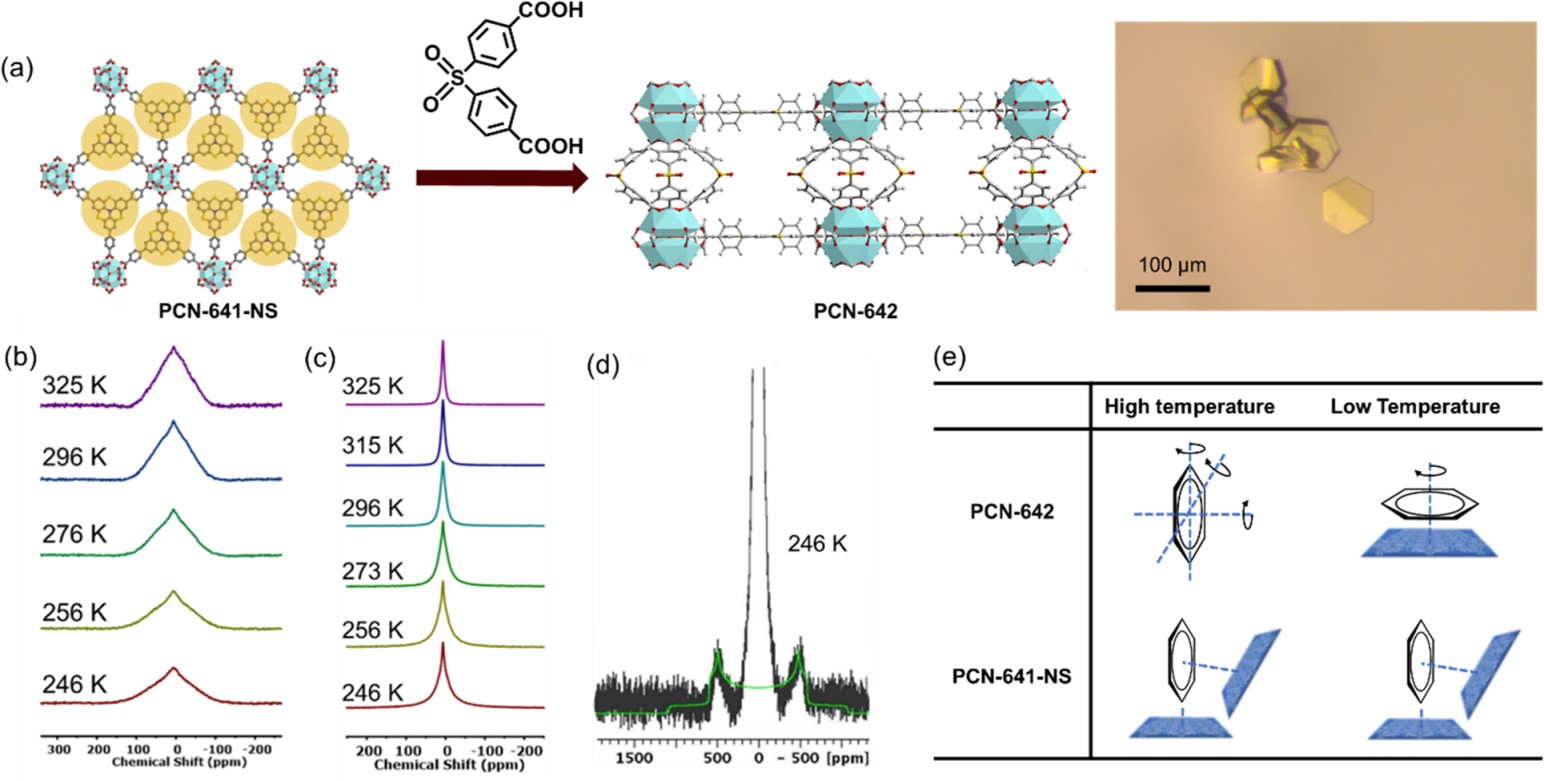
(a) The introduction of the DCDPS ligand to build pillar-layered PCN-642, the single crystal under an optical microscope, is shown. The X-ray single crystal structure is shown in the Supporting Information. (b) Solid-state ^2^H NMR spectra of C_6_D_6_ in PCN-641-NS at the temperature from 325 to 246 K. (c) Solid-state ^2^H NMR spectra of C_6_D_6_ in PCN-642 at the temperature from 325 to 246 K. (d) Solid-state ^2^H NMR spectra of C_6_D_6_ in PCN-642 at 246 K. (e) Proposed rotational motion modes of C_6_D_6_ in the pore space of PCN-642 and PCN-641-NS.

It is important to understand the difference between the interaction of substrate molecules and pores in 3D and 2D MOFs. One premise important to the validity of the comparison between 3D MOFs and 2D MOF nanosheets is that the 3D structures are completely 3D, while PCN-641 is formed by stacked layers of 2D MOFs and does not maintain a fixed layer distance due to the lack of supporting pillar ligands. To tackle this issue, following a previous report to install ligands to form pillar-layered 3D MOFs,^33^ a ditopic pillar ligand, 4,4′-dicarboxylatediphenyl sulfone (DCDPS), was introduced during framework growth and built a pillar-layered 3D MOF, PCN-642 (Figure 6a). Owing to the limited installation ratio and flexibility of DCDPS ligands, PCN-642 is relatively less stable. No permanent porosity was detected after supercritical CO_2_ pore activation, and the one PXRD peak is split into multiple peaks (Figure S18). Instead, single-crystal X-ray diffraction (SCXRD) results of PCN-642 were collected, and the data are provided in Table S2. This 3D analogue of PCN-641-NS provides a background comparison to understand the difference between 2D and 3D host–guest interactions.

Owing to the heterogeneity of the 2D MOF nanosheets, many characterization techniques that require homogeneous dispersion do not apply. Solid-state NMR spectroscopy is a powerful tool to cope with this challenge and sheds light on the host–guest interaction. The phenothiazine core of PCN-641-NS is capable of energy transfer to iodobenzene likely via the π-interaction of the aromatic systems.^34^ Deuterated benzene (C_6_D_6_) can be a probe molecule to differentiate ^2^H from ^1^H in the frameworks. Thus, C_6_D_6_ was doped into the solid materials. The materials were dried to remove the surface-attached molecules for analysis.

To reveal more information on host–guest interaction, the solid-state ^2^H and ^13^C NMR spectra were collected at varied temperatures (Figure 6b-d, Figures S23–S24). The solid Hanh-echo static ^2^H NMR spectrum of benzene-d6 (C_6_D_6_) in PCN-641-NS exhibits a resonance centered at 6.1 ppm and a peak width of 6 kHz at 296 K (Figure 6b). The similar triangle line shape was reported for C_6_D_6_ in microporous aluminum methylphosphonates AlMePO-α and AlMePO-β, where C_6_D_6_ experiences fast (on the NMR time scale) anisotropic flipping reorientations reducing deuterium quadrupolar coupling constants (DQCC).^35^ The formal simulation of the ^2^H signal observed in the NMR spectrum of C_6_D_6_ in PCN-641-NS shows that the signal can be a superposition of the major resonance with DQCC of 7 kHz and the asymmetry parameter (η) of 0.7. The anisotropic motion of C_6_D_6_ in the pores of PCN-641-NS strongly reduces DQCC to 7 kHz versus 180 kHz of static C_6_D_6_. In contrast, C_6_D_6_ in PCN-642 shows a standard Lorentz-shaped line (Figure 6c), whose chemical shift is 6.7 ppm with a line width of 2.8 kHz at 296 K. The smaller line width and Lorentz shape indicate a weaker interaction between C_6_D_6_ and the PCN-642 framework. In the ^13^C NMR spectrum of C_6_D_6_ in PCN-641-NS at 298 K, the carbon resonance is also broadened versus the narrow liquid-like resonance of C_6_D_6_ in PCN-642 (Figure S19). This result supports the quadrupolarity of the deuterium resonance of C_6_D_6_ in PCN-641-NS. It is worth noting that the ^2^H and ^13^C peaks of the adsorbed C_6_D_6_ in PCN-641-NS are slightly high-field shifted (δ_2H_ = 6.1 ppm δ_13C_ = 124 ppm) relative to the sharp peak of C_6_D_6_ in PCN-642 (δ_2H_ = 6.7 ppm, δ_13C_ = 127 ppm), which indicates the stronger shielding and interaction between C_6_D_6_ and the 2D MOF nanosheets.
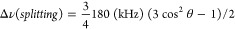
1

The spectral evolution of PCN-642’s ^2^H NMR spectrum manifests a quadrupolar Pake pattern. The Pake pattern can be simulated with a splitting efficiency of 62.8 kHz (Figure 6d). According to the mathematical relationship between the splitting frequency and the coupling constant, the C_6_ rotation where the benzene molecule is parallel to the surface should be calculated by eq 1 with θ = 90°. It yields a 67.5 kHz theoretical splitting efficiency, roughly fitting 62.8 kHz measured by experiment disregarding temperature effect.^36^ The frequency match suggests the parallel adhering of benzene on the pore surface at low temperature. At room temperature, the sharp NMR peak indicates that benzene molecules perform isotropic rotation, manifesting a liquid-like motion in the MOF pores. Interestingly, without evident spectral evolution, PCN-641-NS did not show the same effect in decreasing temperatures. The static Hahn echo ^2^H and ^13^C NMR spectra show consistent broad peaks among the wide temperature range tested. This is largely due to the limited freedom of rotation of benzene molecules in PCN-641-NS pores. The solid-state NMR depicted the motion of benzene in PCN-641-NS and PCN-642’s pores and is summarized in Figure 6e. Even at a high temperature, the strong interaction between the 2D MOF nanosheets and the benzene can confine the adsorbed molecules to anisotropic motion.

TGA-DSC experiments revealed that the evaporation heat of iodobenzene becomes higher in PCN-641-NS compared to PCN-642 when confined in their pore space (Figures S25–S26). The TGA-calculated loading ratio of iodobenzene in PCN-641-NS is 44 wt %, while PCN-642 stores 70 wt %. The TGA-DSC calculated evaporation heats of iodobenzene of PCN-641-NS and PCN-642 are −12.1 and −6.1 mW/g, respectively. The higher heat indicates the more energy input to remove loaded molecules and the stronger confinement by nanosheets.

**Figure 7:**
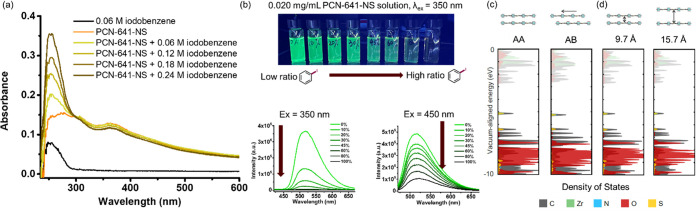
(a) UV–visible absorption spectra of PCN-641-NS (0.020 mg/mL) in a tetrahydrofuran (THF) solution titrated by iodobenzene in aliquots. (b) Fluorescence quenching of PCN-641-NS at increasing ratios of iodobenzene in a THF solution. (c) Computed band structures of PCN-641-NS in AA and AB stacking modes. (d) Computed band structures of PCN-641-NS with interlayer distances of 9.7 and 15.7 Å.

The unique behavior of surface-anchored substrates leads to the formation of an electron-donor–acceptor (EDA) complex in a heterogeneous state. UV–vis studies were conducted to study the energy transfer and adduct formation. Figure 7a shows that iodobenzene’s absorbance λ_max_ in THF shifted from 245 nm (pure iodobenzene) to 255 nm (PCN-641-NS and iodobenzene). This indicates the formation of an EDA complex that has been hardly detected in heterogeneous catalysts and might lead to more efficient energy transfer in photocatalysis. Fluorescence studies showed the quenching of PCN-641-NS with the increasing ratio of iodobenzene (the substrate molecule) in THF solutions under 350 and 450 nm excitation (Figure 7b). This indicates a photoenergy transfer from nanosheets to iodobenzene.

### Band Structure and Catalytic Activity

The single-layer 2D MOF nanosheet-substrate interaction was studied by solid-state NMR, UV–vis, and fluorescence spectroscopy. Moreover, the electron transfer taking place in the nanosheet framework can be explained by the band structure obtained by DFT calculations. The interlayer electron transfer can cause aggregation-induced fluorescence quenching, which results in less efficient photoenergy transfer to substrates. Thus, the aggregation of layers needs to show a minimal effect on the electronic structure of nanosheets to avoid undermining the catalytic efficiency. The simulation of PCN-641-NS and computation of solid-state band gaps were conducted using AA and AB stacking models (Figure 7c). From the introduction of the phenothiazine core (sulfur and carbon), a midgap electronic state emerges with orbital character centered on S, C, and N. This is in accordance with the electron-donor role of phenothiazine-based photocatalysts. The stacking mode does not have an influence on the midgap electronic state, thus not affecting the catalytic activity. Different interlayer distances (9.7 and 15.7 Å) were modeled (Figure 7d) to study the effect on nanosheet electronic structure. No evident variation was observed in the different distances simulated (Figure S33). Supported by the lack of observable correlation of the interlayer stacking mode and distance with the nanosheet’s electronic structure, it can be concluded that the catalytic activity maintains even if aggregation takes place, which is a preferred feature for heterogeneous photocatalysts.

The simulated band structure results also suggest minor interlayer electron transfer. Owing to Zr_6_ cluster’s closed-shell electronic structure, PCN-641-NS is unlikely to conduct electricity in 2D without delocalized charge carriers. In support of this hypothesis, the electric conductivities of PCN-641-NS and PCN-641-NSO are low as tested by a 4-point probe (3.61 × 10^–9^ S/cm and 3.86 × 10^–9^ S/cm, respectively, Figure S31). After the nanosheets are doped with diiodine (I_2_), an ∼10^2^ magnitude conductivity increase was observed (9.00 × 10^–8^ S/cm and 4.28 × 10^–7^ S/cm, respectively), owing to the interlayer electron transfer from the valence band of PCN-641-NSO to the LUMO of I_2_. This increase is more evident on partially oxidized PCN-641-NSO, which can be ascribed to the band enrichment by the introduction of oxygen. Even though it is one of the rare cases of detectable conductivities in Zr-MOFs, their low electric conductivity aligns with the photocatalytic application of this material to keep the photocatalytic efficiency high.

The catalytic performance is expected to improve by going 2D. PCN-641-NS is capable of photoborylation as shown in Table S3 and Figure S29. This reaction was reported in PCC-40 assembled by phenothiazine ligands.^34^ Using iodobenzene and bis(pinacolato)diboron as the starting materials, aryl boronic acid and aryl boronic pinacol ester can be synthesized, which are both reactive substrates for Suzuki coupling to build C–C bonds. A total product yield (including phenylboronic pinacol ester and phenylboronic acid) of 86% at 3 h was observed when PCN-641-NS was utilized in the solvent condition of 5% H_2_O acetonitrile. As proposed in the mechanism (Figure S29d), water plays an important role in protonating tributylamine, thus explaining the decrease in yield when the reaction is performed in pure acetonitrile. In comparison, PCN-641 and PCN-642 crystals were utilized in the same reaction conditions. Their slower reaction kinetics than PCN-641-NS in 5% H_2_O acetonitrile indicates a mass transfer limit (Figure S29b). PCN-641-NS inherits the activity of phenothiazine and leverages it into a heterogeneous catalytic platform with possible recyclability. The recycled PXRD shows that crystallinity is retained with the two major peaks (200) and (020) after catalytic reactions.

**Figure 8:**
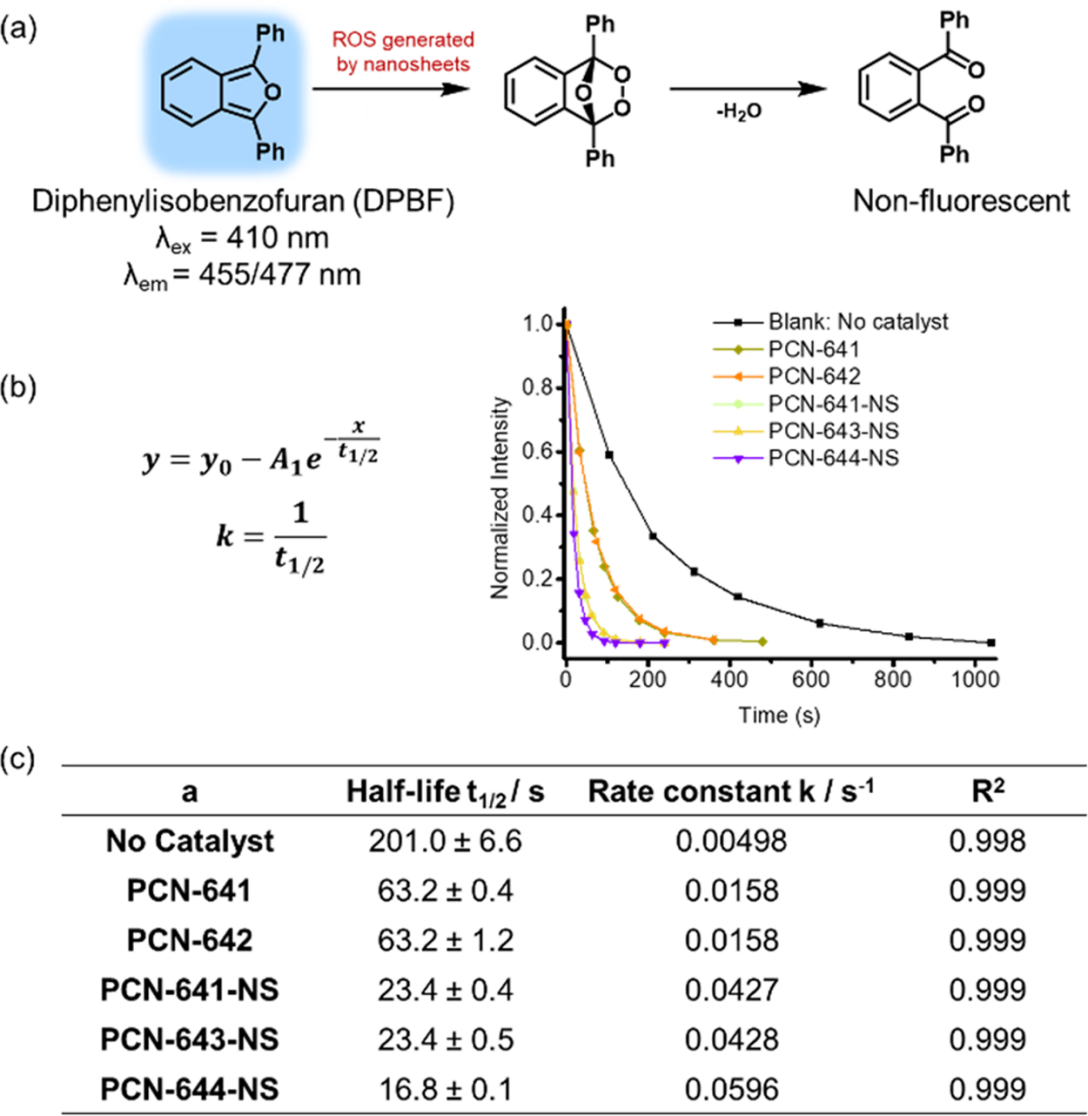
DPBF fluorescence quenching by 2D MOF nanosheet catalyzed-ROS generation. (a) DPBF oxidation by ROS with fluorescence quenching. (b) The first-order reaction kinetics of fluorescence quenching (λ_ex_ = 310 nm, λ_em_ = 455 nm). (c) Calculated reaction lifetimes and rate constants.

A more generic class of photocatalysis is reactive oxygen species (ROS)-assisted oxidation. The generation of ROS can assist multiple oxidative reactions by design. As proved by fluorescence studies, PCN-641-NS can perform photoinduced energy transfer to dioxygen dissolved in solution and produce activated ^1^O_2_. Diphenylisobenzofuran (DPBF) is a fluorescent molecule (λ_ex_ = 410 nm) that is sensitive to singlet oxygen. The oxidation reaction is that the dioxygen inserts into the furan ring, cleaves the furan ring, turns it into a diketone, and diminishes the fluorescence (Figure 8a). Thus, DPBF is selected as a probe molecule: by monitoring the fluorescence quenching, the kinetics of nanosheet-catalyzed ^1^O_2_ formation can be depicted (Figure 8b). PCN-641-NS, PCN-643-NS, PCN-644-NS, PCN-641, and PCN-642 were separately added as photocatalysts (Table S4). The reaction kinetics was collected in Figure S30 and summarized in Figure 8c. A slow base reaction rate was observed in the blank control, where no catalyst was added. This is because DPBF can also act as a photosensitizer to induce energy transfer to the dioxygen and oxidize itself. A higher reaction rate was discovered with the catalyst added. Bulk PCN-641 and PCN-642 are slower than nanosheets, which is in accordance with the hypothesized limited mass transfer rate.

Interestingly, the rate constants of PCN-641-NS and PCN-643-NS are close, while PCN-644-NS’s rate constant is higher than theirs. A possible explanation is the inheritance of the organic photocatalysts’ mechanism into 2D MOF nanosheets. The photoactive centers (phenothiazine, pyrene, and porphyrins) have varied excited state energies and might lead to the difference in the reaction rate.^37,38^ However, the reaction kinetics of nanosheets is fast under the conditions studied. More rigorous photophysics studies might provide more mechanistic insight into the photoactive 2D MOF nanosheets.

## Conclusions

In conclusion, to establish a heterogeneous photocatalytic platform, a synthetic strategy to incorporate photoactive organic molecules into 2D MOF nanosheets via a bottom-up route is demonstrated by PCN-641-NS, PCN-643-NS, and PCN-644-NS. Ultrathin layers of 2D MOF nanosheets were obtained and characterized by electron microscopy and AFM. To compare the host–guest interaction in 3D MOF and 2D MOF nanosheet pores, pillar-layered PCN-642 was synthesized and elaborated by a single crystal structure. A stronger interaction was discovered between PCN-641-NS and guest molecules than the 3D MOF analogue, PCN-642. UV–vis and fluorescence quenching studies indicate the formation of the EDA complex in the heterogeneous phase. Provided the vast catalytic activities of phenothiazine-based organic catalysts, PCN-641-NS was proved efficient in photoborylation and photoinduced ROS generation, and PCN-643-NS and PCN-644-NS demonstrated catalytic activity in the reaction systems. This work provides a synthetic perspective for the heterogenization of organic photocatalysts and a generic method to study their host–guest interactions and catalytic activities.

## Supporting Information Available

Experimental procedures. Materials and instrumentation. The synthesis of H_3_**L1**, H_4_**L2**, and H_4_**L3**. The syntheses of PCN-641, PCN-642, PCN-641-NS, PCN-643-NS, and PCN-644-NS. Electron microscopy (SEM and TEM) procedure and results. Atomic force microscopy (AFM) procedure and results. X-ray diffraction results (single-crystal for PCN-642 and powder for all the samples mentioned in the manuscript). Solid-state NMR results (^13^C NMR). Gas uptake analysis (procedure and results). Thermogravimetric analysis (PCN-641-NS and PCN-642). Photoreactions (procedures and results of photoborylation and photoinduced ROS generation). Electric conductivities. Band structure simulations (PDF)
