# Supplementary material for: Integrating Photoactive Ligands into Crystalline Ultrathin 2D Metal–Organic Framework Nanosheets for Efficient Photoinduced Energy Transfer
Source: J Am Chem Soc. 2024 Jan 3;146(2):1491–500. doi: 10.1021/jacs.3c10917 (PMC10863068; doi:10.1021/jacs.3c10917)
Supplement: Supplementary file 1 — ja3c10917_si_001.pdf [file ja3c10917_si_001.pdf]

Supporting Information

Integrating Photoactive Ligands into Crystalline Ultra-thin 2D Metal-Organic Framework Nanosheets for Efficient Photo-induced Energy Transfer

Hengyu Lin, Yihao Yang, Brian G. Diamond, Tian-Hao Yan, Vladimir I. Bakhmutov, Kelechi W. Festus, Peiyu Cai, Zhifeng Xiao, Mingwan Leng, Ibukun Afolabi, Gregory S. Day, Lei Fang,* Christopher H. Hendon,* and Hong-Cai Zhou*

Contents

Section 1. Materials and Instrumentation 3
Section 2. The synthesis of ligand H_3**L1**, H_4**L2**, H_4**L3** 4
Section 3. The synthesis of PCN-641 5
Section 4. The synthesis of PCN-642 6
Section 5. The syntheses of PCN-641-NS, PCN-643-NS, and PCN-644-NS 6
Section 6. Electron Microscopy 7
6.1 Scanning Electron Microscopy (SEM) 7
6.2 Transmission Electron Microscopy (TEM) 9
Section 7. X-ray Diffraction 10
7.1 Powder X-ray Diffraction 10
7.2 Single Crystal X-ray Diffraction 12
Section 8. Atomic Force Microscopy (AFM) 14
Section 9. Gas Uptake Analysis 14
Section 10. Solid-state NMR (ssNMR) 15
Section 11. Thermogravimetric analysis 17
Section 12. Photoreactions 18
12.1 Photo-borylation 18
12.2 Photo-induced ROS generation 19
Section 13. Electric Conductivities 21
Section 14. Band Structure Simulation 21

Section 1. Materials and Instrumentation

(N-phenyl)phenothiazine (PTH), zirconium tetrachloride, iodobenzene, bis(pinacolato) diboron, N, N-dimethylformamide (DMF), dimethyl sulfoxide (DMSO), acetone, methanol, ethanol were purchased from Matrix Scientific, TCI, Ambeed, and Supelco Omnisolv.

X-ray Powder Diffraction was carried out on Quest ECO Molybdemum source Photon II detector. Thermogravimetric analyses (TGA) were carried out on METTLER TOLEDO TGA/DSC 1 thermogravimetric analyzer from room temperature to 800 ^{o}C at a ramp rate of 2 ^{o}C/min in a flowing nitrogen atmosphere. Nitrogen adsorption measurements were conducted on Micromeritics ASAP 2020 at a temperature of 77.3 K (liquid nitrogen). Nuclear magnetic resonance (NMR) spectra were collected on AVANCE NEO 400 (TANGO and WALTZ). ESI-Mass spectra were collected on Thermo Scientific Q Exactive Focus. The UV-Visible electronic absorption spectra were collected on Shimadzu UV-2450 Spectrophotometer and Hitachi U-4100 Spectrophotometer. The fluorescence spectra (excitation, emission, and lifetime) were collected on PTI QuantaMaster series spectrofluorometer and Horiba Fluorescence Spectrophotometer. High-performance liquid chromatography (HPLC) was carried out on a Horiba LC system.

Section 2. The synthesis of ligand H_3**L1**, H_4**L2**, H_4**L3**

The synthesis of ligand H_3**L1** is conducted following the reported procedure in supporting information of Lin et al.'s paper.[1] Ligand H_3**L2** synthesized by the reported synthetic procedures for H_4TBAPy and ligand H_3**L3** was synthesized following the reported synthetic procedures of TCPP.[2-3] The ligand structures are summarized in Figure S1.

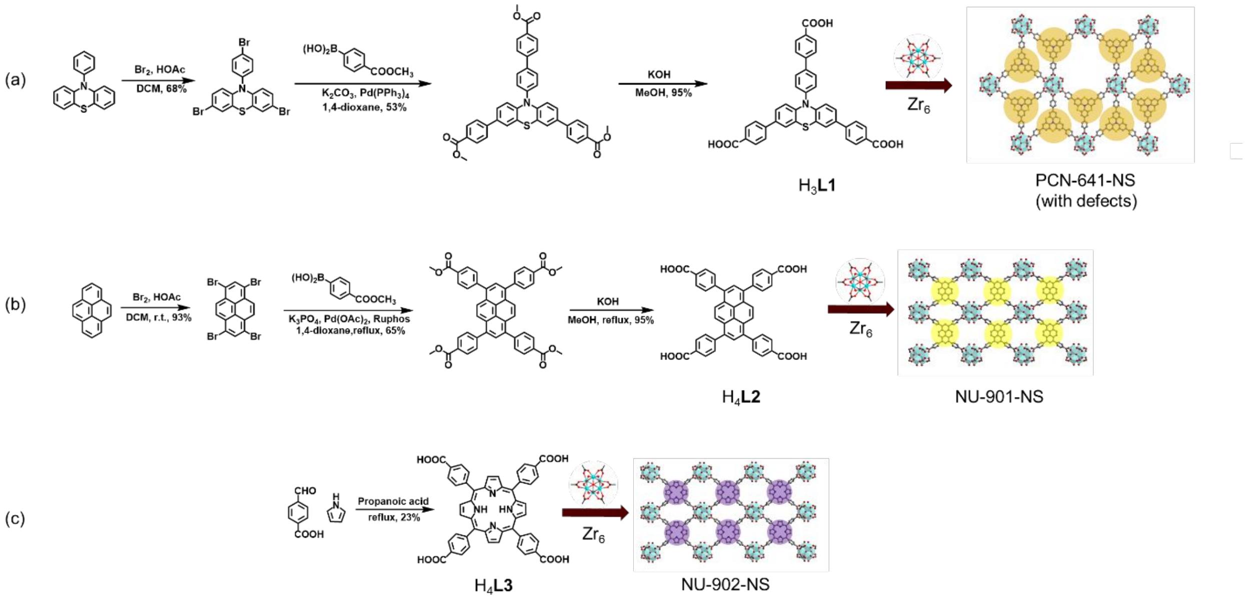

Figure S1. Synthetic schemes of ligands H_3**L1**,[1] H_4**L2**,[3] and H_4**L3**.[3] Synthetic procedures are adopted from the references cited.

Section 3. The synthesis of PCN-641

By formic acid (FA): 1 mL of pre-dissolved *N,N*-dimethylformamide (DMF) H_3**L1** (20 mg/mL, 0.031 mmol/mL) solution was charged into a 4-mL vial with a Teflon cap. 600 μL formic acid (FA) was charged into the vial. The yellow solution was sonicated for 5 minutes.

By trifluoroacetic acid (TFA):1 mL pre-dissolved DMF zirconium tetrachloride ($ZrCl_4$, 40 mg/mL, 0.17 mmol/mL) solution was charged into the 4-mL vial. Thus, a vial contains 20 mg $ZrCl_4$, 10 mg H_3**L1**, 2 mL DMF, and 100 μL TFA. The mixture was sonicated for another 5 minutes to yield a clear yellow synthetic solution. The vial charged with the synthesis solution was heated to 120 ^{o}C for 24 hours. Yellow crystals precipitated at the bottom of the 4-mL vial. The crystals were collected via centrifuge and activated by solvent exchange. The activation process is discussed in Section 9.

Section 4. The synthesis of PCN-642

1.5 mL of pre-dissolved *N,N*-dimethylformamide (DMF) H_3**L1** (20 mg/mL, 0.031 mmol/mL) solution was charged into a 4-mL vial with a Teflon cap. 1.5 mL pre-dissolved DMF zirconium tetrachloride ($ZrCl_4$, 40 mg/mL, 0.17 mmol/mL) solution was charged into the same 4-mL vial. The mixture was sonicated to yield a clear yellow solution and heated at 85 ^{o}C for 1 hour. 4,4'-sulfonyldibenzoic acid (DCDPS, 15 mg, 0.049 mmol) and benzoic acid (BA, 300 mg, 2.46 mmol) were added into the solution. The synthetic mixture was sonicated for a small period to allow for the dissolution of the added chemicals and immediately heated to 120 ^{o}C for 48 hrs. The crystals were collected by centrifuge and activated through solvent exchange.

Section 5. The syntheses of PCN-641-NS, PCN-643-NS, PCN-644-NS, and PCN-641-NSO

Top-down synthetic steps for PCN-641-NS: 3 mg of PCN-641 was added into 3 mL water in a 4-mL glass vial. The vial is sonicated for 20 min, and then cooled for 20 min to avoid the temperature rise of the solution. The total sonication time amounts to 1 hour. After centrifuging the solution at 6,000 rpm for 30 min, the yellow precipitate un-dispersed PCN-641 was collected with well-dispersed solution of exfoliated PCN-641-NS. The solution was directly used as the sample for "top-down" synthesized PCN-641-NS in SEM and TEM analysis. Only a small amount of PCN-641 was successfully exfoliated. Thus, the yield of top-down method was not calculated.

Bottom-up synthetic steps for PCN-641-NS: 1.5 mL of pre-dissolved *N,N*-dimethylformamide (DMF) H_3**L1** (21.6 mg/mL, 0.033 mmol/mL) solution was charged into a 4-mL vial with a Teflon cap. 1.5 mL of pre-dissolved DMF zirconium tetrachloride ($ZrCl_4$, 12 mg/mL, 0.051 mmol/mL) solution was charged into the same 4-mL vial. The mixture was sonicated to yield a clear yellow solution. Modulators and varying amounts of water were added to the solution. The synthetic mixture was sonicated for 10 min and heated at 120 ^{o}C for 48 hours. Condition screening is reported in Table S1.

Synthetic steps for PCN-643-NS and PCN-644-NS: the synthetic procedures of PCN-641-NS were adopted with varied ligands and monotopic carboxylic acid. The rest of the conditions were kept the same. Condition screening is reported in Table S1. PCN-643-NS yielded the best crystallinity with acetic acid as the modulator and PCN-644-NS with formic acid.

Bottom-up synthetic steps for PCN-641-NSO: 1.5 mL of pre-dissolved *N,N*-dimethylformamide (DMF) H_3**L1** (21.6 mg/mL, 0.033 mmol/mL) solution was charged into a 4-mL vial with a Teflon cap. The solution was bubbled with air for 1 hour. 1.5 mL of pre-dissolved DMF zirconium tetrachloride ($ZrCl_4$, 12 mg/mL, 0.051 mmol/mL) solution was charged into the same 4-mL vial. The mixture was sonicated to yield a clear yellow solution. Modulators and varying amounts of water were added to the solution. The synthetic mixture was sonicated for 10 min and heated at 120 ^{o}C for 48 hours.

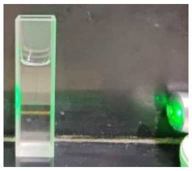

blank comparison

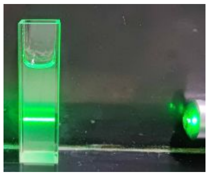

Tyndall effect

Figure S2. Tyndall effect of PCN-641-NS solution against blank solvent.

Top-down synthesis of nanosheets: 3 mg of bulk PCN-641 was added into 3 mL methanol in a 4-mL glass vial. The vial is sonicated for 20 min, and then cooled for 20 min to avoid the temperature rise of the solution. The total sonication time amounts to 1 hour. After centrifuging the solution at 6,000 rpm for 30 min, the yellow precipitate un-dispersed PCN-641 was collected with dispersed solution of exfoliated PCN-641-NS. The solution was directly used as the sample for “top-down” synthesized PCN-641-NS in SEM, TEM, and AFM analysis.

3 mg of bottom-up synthesized PCN-641-NS was added into 3 mL methanol in a 4-mL glass vial. The vial is sonicated for 20 min, followed by centrifuging at 6,000 rpm for 30 min. A dispersed solution of PCN-641-NS was obtained with no precipitate. The solution was used as the sample for SEM and TEM analyses.

Table S1. Synthetic conditions of PCN-641-NS

Nanosheet	Modulator	Modulator Amount (Volume/mass)	Water Volume/ µL
PCN-641-NS-FA1	Formic acid	500 µL	0
PCN-641-NS-FA2			35
PCN-641-NS-FA3			100
PCN-641-NS-TFA1	Trifluoroacetic acid	100 µL	0
PCN-641-NS-TFA2			35
PCN-641-NS-TFA3			100
PCN-641-NS-AA1	Acetic acid	300 µL	0
PCN-641-NS-AA2			35
PCN-641-NS-AA3			100
PCN-641-NS-PA1	Propanoic acid	300 µL	0
PCN-641-NS-PA2			35
PCN-641-NS-PA3			100
PCN-641-NS-CA1	Caproic acid	300 µL	0
PCN-641-NS-CA2			35
PCN-641-NS-CA3			100
PCN-641-NS-BA1	Benzoic acid	300 mg	0
PCN-641-NS-BA2			35
PCN-641-NS-BA3			100
Nanosheet	Modulator	Modulator Amount (Volume/mass)	Water Volume/ µL
PCN-643-NS-FA1	Formic acid	500 µL	0
PCN-643-NS-FA2			35
PCN-643-NS-FA3			100
PCN-643-NS-TFA1	Trifluoroacetic acid	100 µL	0
PCN-643-NS-TFA2			35
PCN-643-NS-TFA3			100
PCN-643-NS-AA1	Acetic acid	300 µL	0
PCN-643-NS-AA2			35
PCN-643-NS-AA3			100
PCN-643-NS-PA1	Propanoic acid	300 µL	0
PCN-643-NS-PA2			35
PCN-643-NS-PA3			100
PCN-643-NS-CA1	Caproic acid	300 µL	0
PCN-643-NS-CA2			35
PCN-643-NS-CA3			100
PCN-643-NS-BA1	Benzoic acid	300 mg	0
PCN-643-NS-BA2			35
PCN-643-NS-BA3			100
Nanosheet	Modulator	Modulator Amount (Volume/mass)	Water Volume/ µL
PCN-644-NS-FA1	Formic acid	500 µL	0
PCN-644-NS-FA2			35
PCN-644-NS-FA3			100
PCN-644-NS-TFA1	Trifluoroacetic acid	100 µL	0
PCN-644-NS-TFA2			35

PCN-644-NS-TFA3			100
PCN-644-NS-AA1	Acetic acid	300 μL	0
PCN-644-NS-AA2			35
PCN-644-NS-AA3			100
PCN-644-NS-PA1	Propanoic acid	300 μL	0
PCN-644-NS-PA2			35
PCN-644-NS-PA3			100
PCN-644-NS-CA1	Caproic acid	300 μL	0
PCN-644-NS-CA2			35
PCN-644-NS-CA3			100
PCN-644-NS-BA1	Benzoic acid	300 mg	0
PCN-644-NS-BA2			35
PCN-644-NS-BA3			100

PCN-641-NS-FA3, PCN-643-NS-AA3, and PCN-644-NS-FA3 showed the best crystallinity and were used to obtain the SEM, TEM, and AFM results for bottom-up synthesized nanosheets shown in the manuscript.

Section 6. Electron Microscopy

6.1 Scanning Electron Microscopy (SEM)

SEM was performed on the nanosheets utilizing an FEI Quanta 600 field emission scanning electron microscope (FE-SEM). PCN-641-NS synthesized under different conditions were summarized. The modulators (monotopic carboxylic acids) and the water concentration were adjusted and summarized in Table S1.

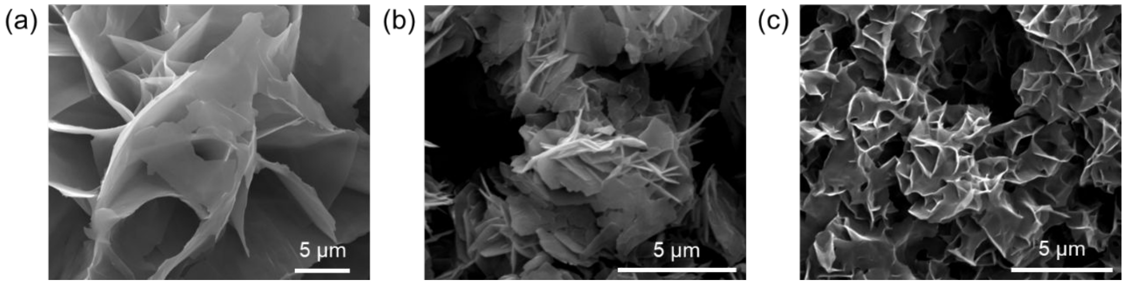

Figure S3. SEM images of (a) PCN-641-NS-FA1, (b) PCN-641-NS-FA2 and (c) PCN-641-NS-FA3.

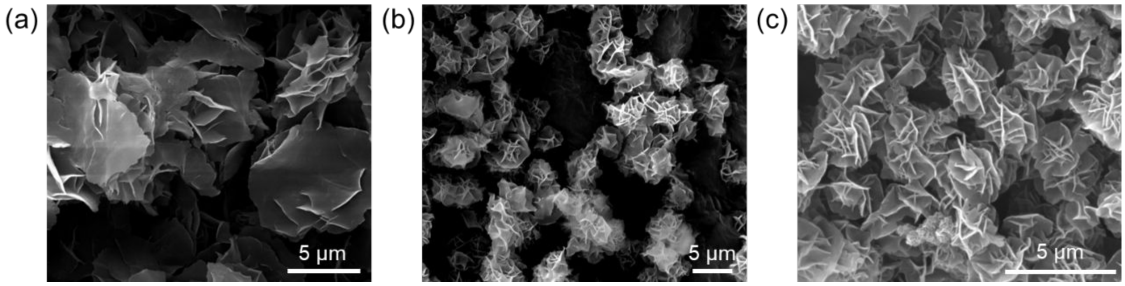

Figure S4. SEM images of (a) PCN-641-NS-TFA1, (b) PCN-641-NS-TFA2 and (c) PCN-641-NS-TFA3.

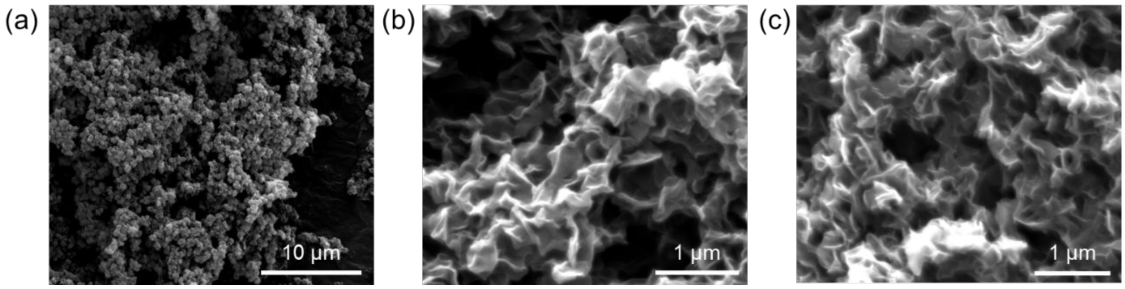

Figure S5. SEM images of (a) PCN-641-NS-AA1, (b) PCN-641-NS-AA2 and (c) PCN-641-NS-AA3.

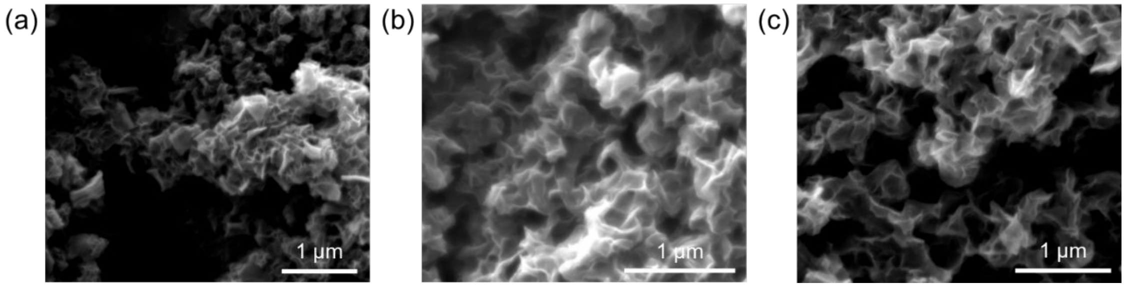

Figure S6. SEM images of (a) PCN-641-NS-PA1, (b) PCN-641-NS-PA2 and (c) PCN-641-NS-PA3.

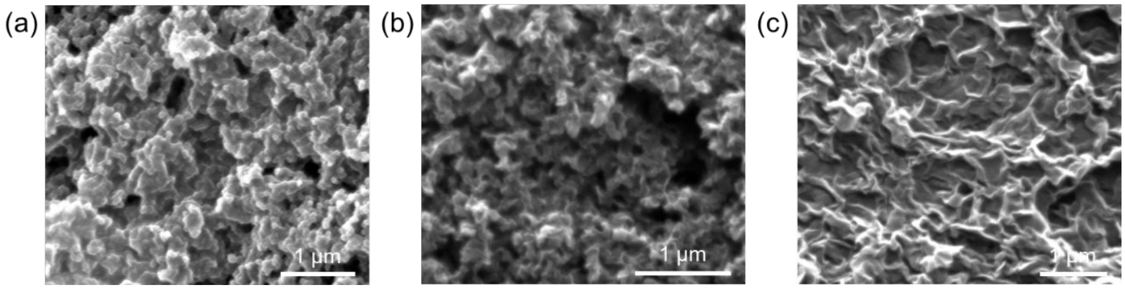

Figure S7. SEM images of (a) PCN-641-NS-CA1, (b) PCN-641-NS-CA2 and (c) PCN-641-NS-CA3.

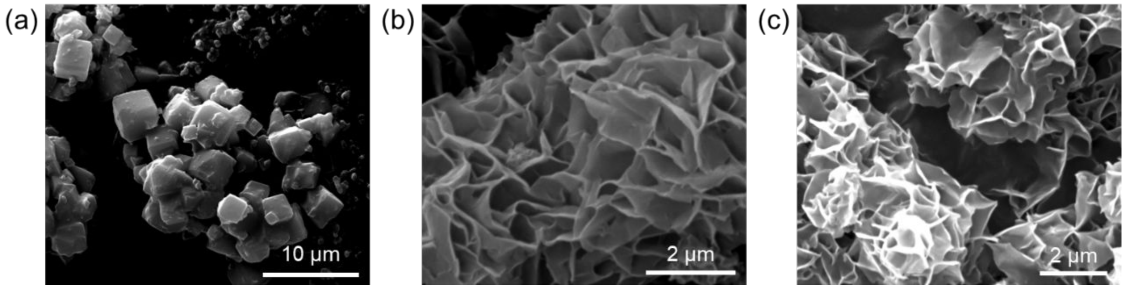

Figure S8. SEM images of (a) PCN-641-NS-BA1, (b) PCN-641-NS-BA2 and (c) PCN-641-NS-BA3.

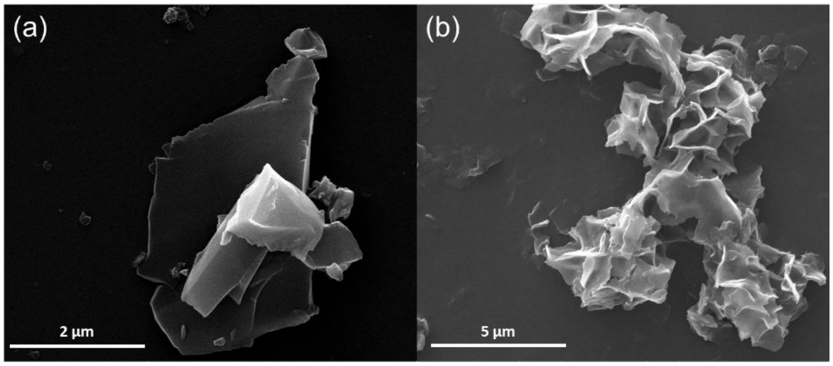

Figure S9. SEM images of (a) PCN-643-NS and (b) PCN-644-NS.

6.2 Transmission Electron Microscopy (TEM)

TEM was performed on the nanosheets utilizing an FEI Tecnai G2 F20 ST field emission transmission electron microscope (FE-TEM), operated at an accelerating voltage of 200 kV.

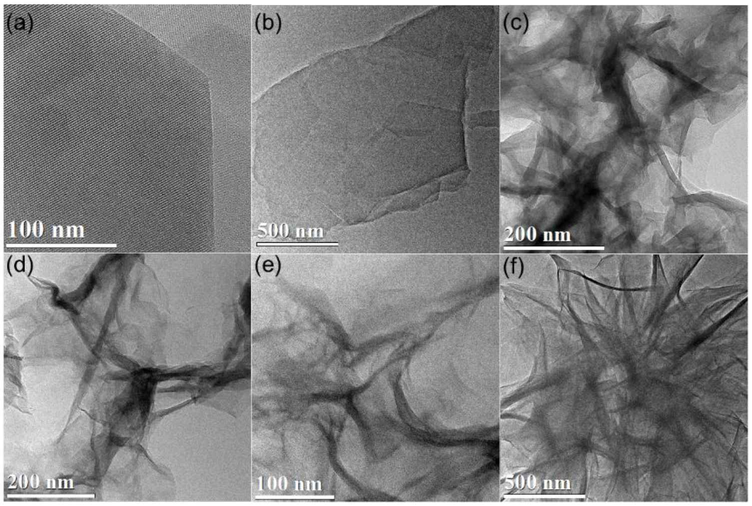

Figure S10. TEM images of (a) PCN-641-NS-FA2, (b) PCN-641-NS-TFA1, (c) PCN-641-NS-AA3, (d) PCN-641-NS-PA3, (e) PCN-641-NS-CA3, and (d) PCN-641-NS-BA3

As can be seen from the SEM and TEM images, PCN-641-NS-FA series yielded the best crystalline nanosheets with uniform thickness. With increasing water concentration, the size of the nanosheet decreases (Figure S3). PCN-641-NS-TFA series also yielded crystalline nanosheets with relatively lower crystallinity (Figure S4b, less clear lattice arrays) compared to PCN-641-NS-FA series with more multi-layer sheets. PCN-641-NS-AA, -PA, -CA, and -BA series show reduced nanosheet crystallinity and other morphologies (Figure S1a, Figure S1a, and Figure S1a). TEM images reveal that thin MOF films were formed. However, due to stronger axial linker interaction (hydrophobic interaction, etc.), the films showed "wet paper" like morphology with reduced crystallinity. Among the PCN-641-NS-FA series, PCN-641-NS-FA2 showed the best crystallinity under SEM and TEM. Thus, it is selected to be the major target of the studies. The "PCN-641-NS" in the main text and other sections of the work refers to PCN-641-NS-FA2.

Section 7. X-ray Diffraction

7.1 Powder X-ray Diffraction

Powder X-ray diffraction patterns were collected on a Bruker D8 Advance ECO powder diffractometer. The data were collected from 2-40°. PCN-641-NS synthesized by different conditions, PCN-642, PCN-643-NS, and PCN-644-NS were tested.

The PXRD result was indexed in Jade to find the miller indices of the two major peaks being (200) and (020). Rietveld refinement of PCN-641-NS-FA was launched in Material Studio. R_{wp} can reach 6.74% with the AB packing mode. The cif file is attached separately.

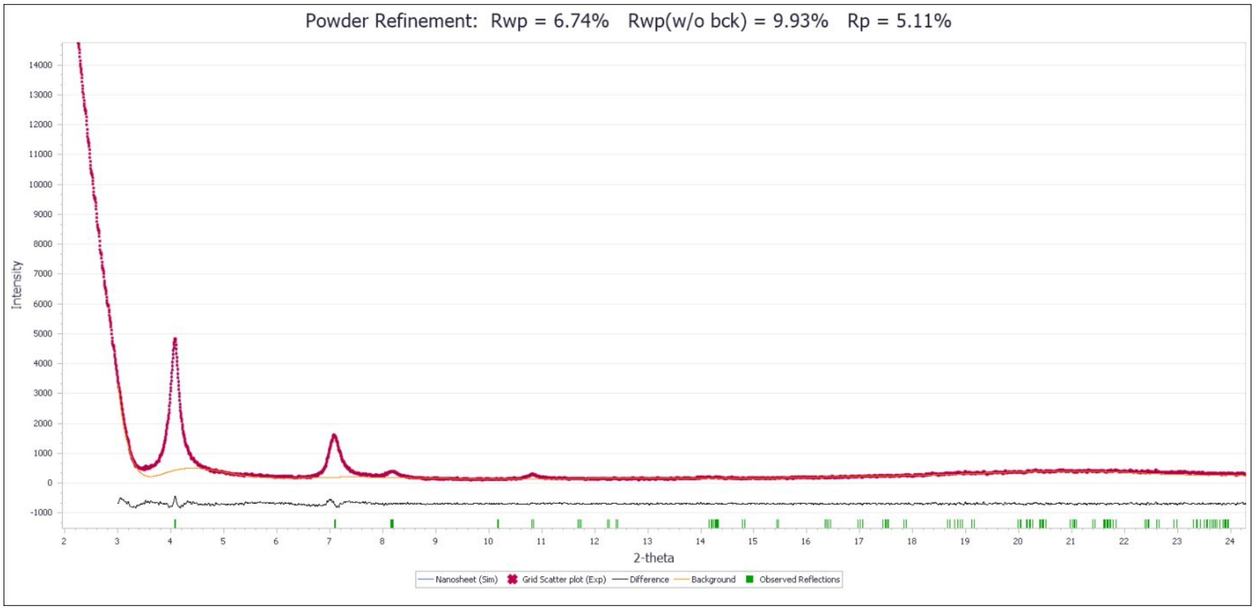

Figure S11. Rietveld refinement of PCN-641-NS-FA.

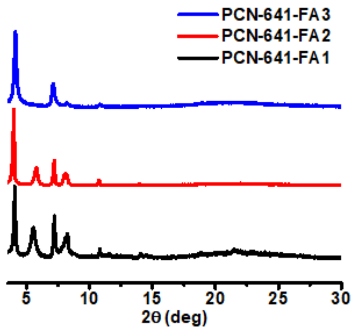

Figure S12. Powder X-ray diffraction patterns of PCN-641-NS-FA series

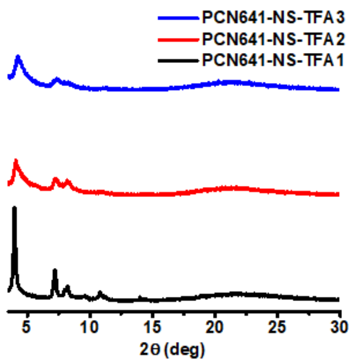

Figure S13. Powder X-ray diffraction patterns of PCN-641-NS-TFA series

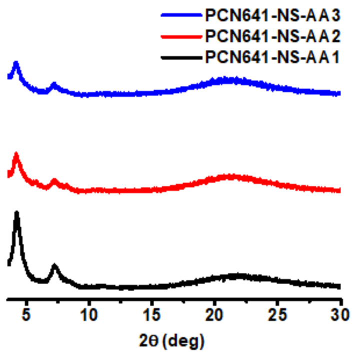

Figure S14. Powder X-ray diffraction patterns of PCN-641-NS-AA series

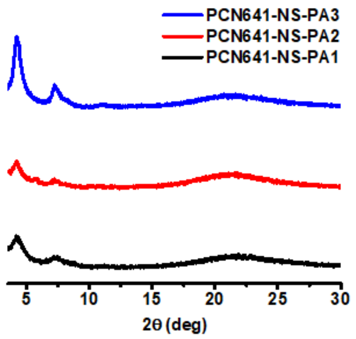

Figure S15. Powder X-ray diffraction patterns of PCN-641-NS-PA series

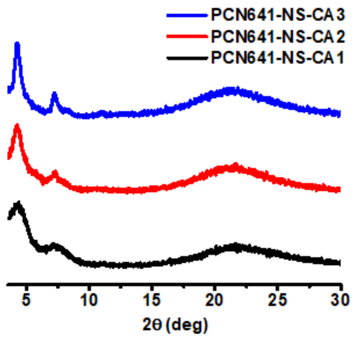

Figure S16. Powder X-ray diffraction patterns of PCN-641-NS-CA series

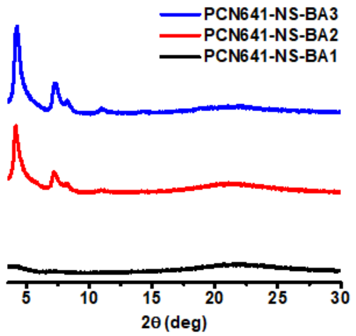

Figure S17. Powder X-ray diffraction patterns of PCN-641-NS-BA series

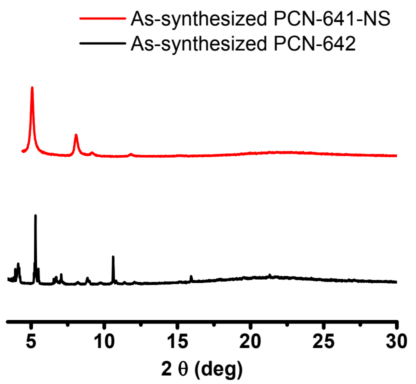

Figure S18. PXRD comparison of PCN-641-NS and PCN-642.

7.2 Single Crystal X-ray Diffraction

Single crystal X-ray diffraction was adopted to determine the crystal structure of pillar-layer PCN-642. Large single crystals suitable for diffraction were obtained following the procedure included in the synthesis. The single crystals were taken from the mother liquor without further treatment. They were then transferred to oil and mounted onto a loop for single-crystal X-ray data collection. The diffraction was measured on a Bruker D8 Venture diffractometer equipped with a Cu Kα sealed-tube X-ray source (λ = 1.54178 Å, graphite monochromated) at 110 K. The structures were solved utilizing direct methods using SHELXT and refined by full matrix least-squares on F^2 using SHELXL software.[4] All non-hydrogen atoms were refined with anisotropic displacement parameters. The hydrogen atoms were located at geometrically calculated positions and refined by riding. The presence of hydrogen atoms on the cluster was calculated by charge balance. The free solvent molecules were highly disordered within the MOF structures and attempts to locate and refine the solvent peaks were unsuccessful. The diffused electron densities resulting from these solvent molecules were removed using the SQUEEZE routine of PLATON. The structures were then refined again using the data generated.[5] It should be noted that the occupancy ratio of pillar ligand, DCDPS, is set to be 0.1 due to the relatively low installation into the crystal. The sulfur in PTH ligand has 3 equal locations with an occupancy of 1/3 according to the C_6 rotation axis in the crystal structure.

Table S2. Crystal data and structural refinement parameters of PCN-642

Identification code	PCN-642
CCDC number	2298059
Empirical formula	$C_{97.2}H_{40.8}N_2O_{27.2}S_{2.6}Zr_6$
Formula weight	2302.39
Temperature/K	110
Crystal system	hexagonal
Space group	P6/mmm
a/Å	24.872(4)
b/Å	24.872(4)
c/Å	14.688(7)
α/°	90
β/°	90
γ/°	120
Volume/Å^3	7869(5)
Z	1
ρ_{calc}g/cm^3	0.486
μ/mm^{-1}	1.933
F(000)	1137.0
Crystal size/mm^3	0.1 × 0.1 × 0.1
Radiation	CuKα (λ = 1.54178)
2Θ range for data collection/°	7.284 to 61.718
Index ranges	$-16 \leq h \leq 14$, $-16 \leq k \leq 12$, $-9 \leq l \leq 9$
Reflections collected	5176
Independent reflections	532 [R_{int} = 0.0891, R_{sigma} = 0.0281]
Data/restraints/parameters	532/152/122
Goodness-of-fit on F^2	2.857
Final R indexes [I>=2σ (I)]	R_1 = 0.2277, wR_2 = 0.5873
Final R indexes [all data]	R_1 = 0.2550, wR_2 = 0.6143
Largest diff. peak/hole / e Å^{-3}	0.56/-0.75

Section 8. Atomic Force Microscopy (AFM)

Stock solutions of PCN-641-NS, PCN-643-NS, and PCN-644-NS were made around 1 mg/mL in acetone. The solutions were diluted by 1000 times to obtain a concentration around 1 μg/mL. The diluted solutions were cast on the matrix plate. One drop was added and evaporated to dry.

The prepared sample was tested by Bruker Dimension Icon AFM yielding high-resolution topographical information images via tapping mode.

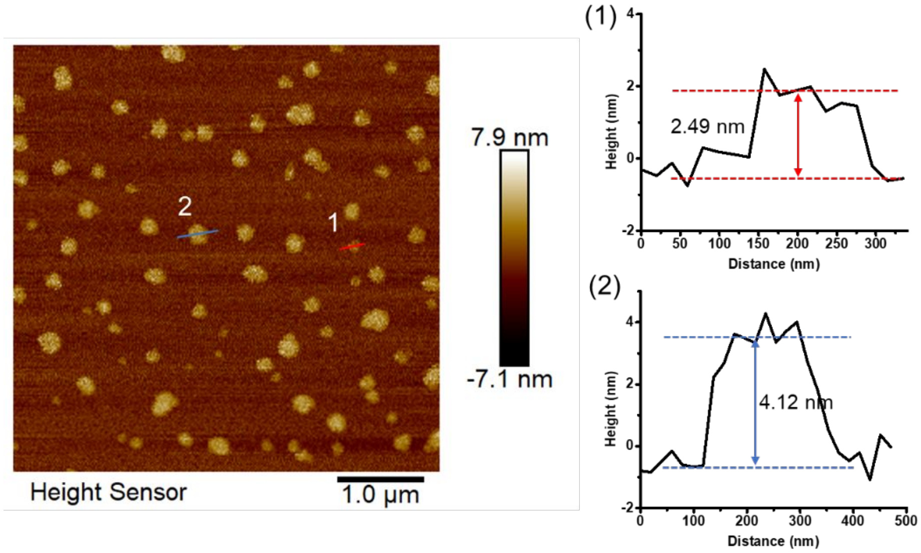

Figure S19. AFM image of PCN-643-NS

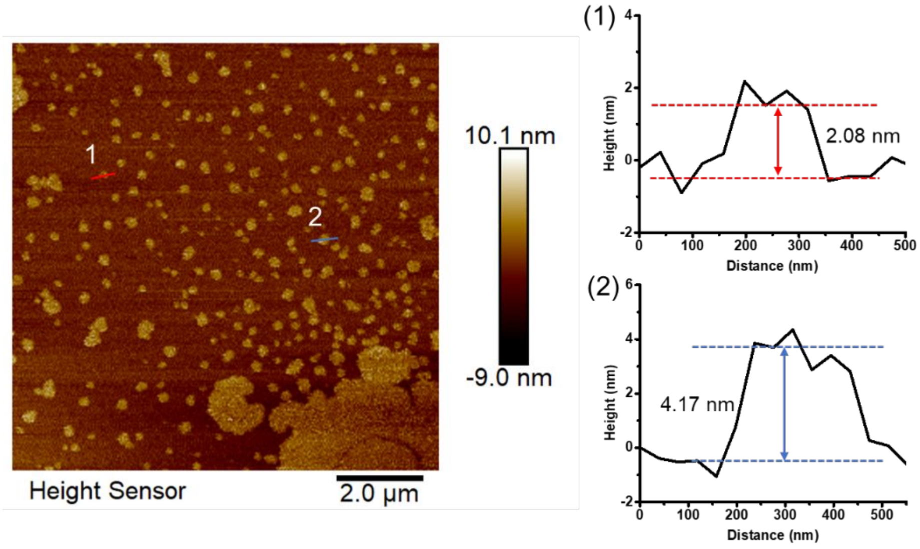

Figure S20. AFM image of PCN-644-NS

Section 9. Gas Uptake Analysis

The gas uptake isotherms were collected on Micromeritics ASAP 2020 surface area and pore size analyzers. Prior to isotherm collection, the as-synthesized samples were washed 5 times by acetone. The solution was centrifuged, and the supernatant was decanted before the next washing solvent was charged. After the final wash, the wet solid samples were left in the open air under darkness for 24 hours to evaporate the acetone. Afterwards, the samples were transferred into BET sample tubes and activated under vacuum at 100 °C for 20 hours.

The N_2 sorption isotherms were collected at 77 K in liquid N_2 bath. BET surface area analysis was conducted on MicroActive software within the range 0-0.15 p/p_0. The pore size distribution was analyzed on MicroActive through DFT calculation method.

PCN-642 didn't show permanent porosity after vacuum activation. Supercritical CO_2 activation was conducted still without the detection of permanent porosity, possibly due to the large pore space and its collapsing during activation.

PCN-641-NS and PCN-641's N_2 uptakes were tested to be 257 cm^3/g and 156 cm^3/g, with the calculated BET surface area being 802 m^2/g and 504 m^2/g, respectively. DFT calculation of pore size distribution was performed. PCN-641-NS show distinctive pore width at 8-9 Å, 10-12 Å, and 18-19 Å, which refer to the in-plane nanosheet pore, out-of-plane nanosheet pore, and missing linker defective pore. Large pores in 30-40 Å are detected in PCN-641-NS and PCN-641, presumably caused by the nanosheet packing.

The N_2 uptake of PCN-643-NS is 395 cm^3/g, while PCN-644-NS is 425 cm^3/g. The BET surface area of PCN-643-NS is calculated to be 573 cm^2/g, and PCN-644-NS is 407 cm^3/g. DFT pore size was calculated for the nanosheets. PCN-643-NS shows two peaks at 12.5 and 14.6 Å, which correspond to the two pores in the nanosheet plane. PCN-644-NS shows a sharp peak at 7.3 Å, and a peak with shoulder at 11-15 Å. They correspond to the inter-layer width of the nanosheet and the pores in the nanosheet plane.

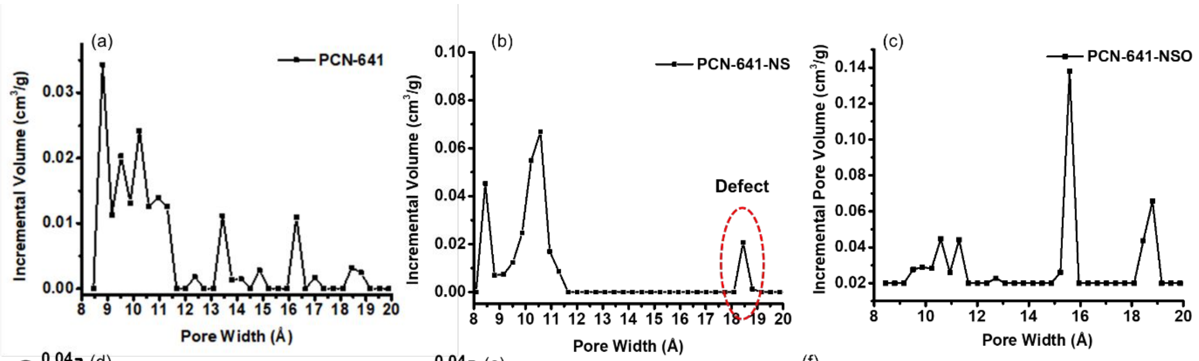

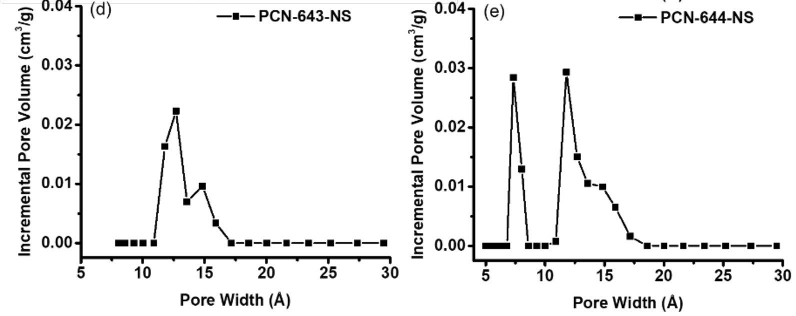

(f)

Item	Details
Geometry	Cylinder
DFT model	N_2 – cylindrical pores – oxide surface
Pressure range	All points measured in the 0 – 0.95 P/P_0 range

Figure S21. DFT calculated micropore size distribution of (a) PCN-641, (b) PCN-641-NS, (c) PCN-641-NSO, (d) PCN-643-NS, and (e) PCN-644-NS.

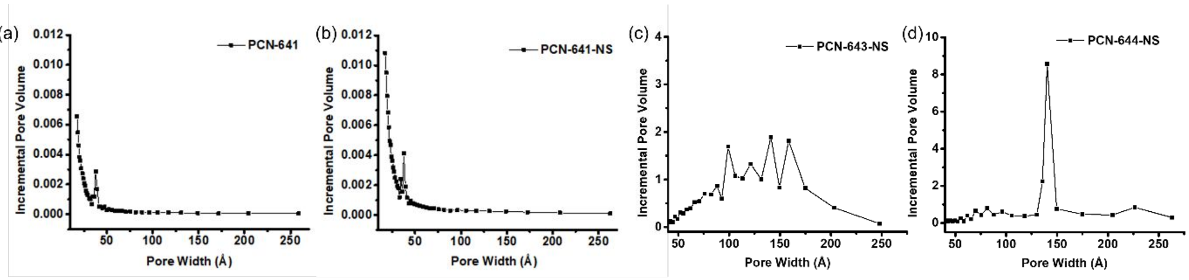

Figure S22. BJH desorption calculated mesopore size distribution of (a) PCN-641, (b) PCN-641-NS, (c) PCN-643-NS, and (d) PCN-644-NS. (Incremental pore volume unit: cm^3/g)

Section 10. Solid-state NMR (ssNMR)

The solid-state NMR spectra of C_6D_6 in PCN-641-NS and PCN-642 were collected on (the instrument). The solid samples were submerged in benzene-d_6 and dried under vacuum to remove the liquid benzene-d_6 and the benzene-d_6 adsorbed on the surface of the solid, leaving only the benzene-d_6 adsorbed in the pores.

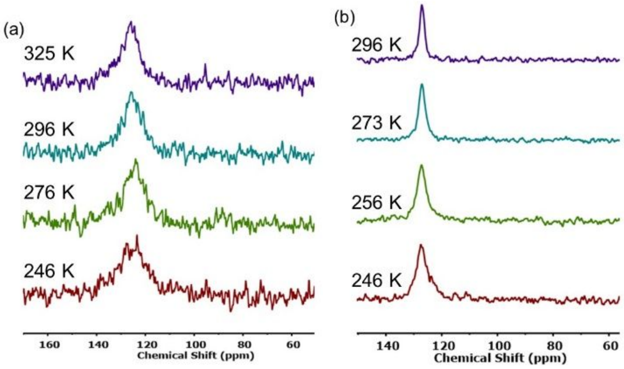

Figure S23. Static ^{13}C solid Hanh-echo NMR spectra of benzene-d_6 adsorbed in (a) PCN-641-NS and (b) PCN-642

It can be seen from X-ray diffraction, structure simulation, SEM, and TEM studies that the pore sizes/interlayer distances are evidently larger (~15 Å). In Figure 5c, the deuterium resonance of C_6D_6 in PCN-642 at 296 K and 274 K is narrow and liquid-like corresponding to fast isotropic reorientations. It should be noted that the line-width remains small (1100 Hz) even at 256 K and is lower than the melting point of C_6D_6 (~274.4 K). When cooled to 246 K, the spectrum evolves an additional low-intensity quadrupolar resonance. The simulation (Figure 5c) leads to a DQCC of 88 kHz ($\eta = 0.1$). The small (close to zero) asymmetrical parameter η indicates the symmetry of this motion. The quadrupolar coupling constant characterizes the C_6D_6 as experiencing the reported fast in-plane C_6 rotation as shown in Figure 5e. The ^{13}C NMR spectra of C_6D_6 in PCN-642 show the narrow liquid-like resonance at 296 K and 273 K with δ = 127 ppm and only at 246 K this signal is slightly broadened in accordance with the ^2H NMR spectra. On the other hand, C_6D_6 in PCN-641-NS is more confined due to the larger asymmetrical parameter and the broader peak width observed.

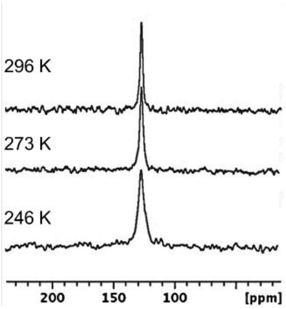

Figure S24. The static ^{13}C one-pulse NMR spectra of C_6D_6 in PCN-642 at 296 K, 273 K, and 246 K.

Section 11. Thermogravimetric analysis

Thermogravimetric analysis (TGA) and differential scanning calorimetry were performed on PCN-641-NS, PCN-642, and corresponding iodobenzene soaked samples. The temperature was raised from room temperature to 80 °C. The weight variation started recording when temperature reached 80 °C and became steady after 9 min. The heat flow starting from room temperature was recorded. The weight variation indicates the mass of iodobenzene evaporated. The corresponding integral of heat flow was calculated during temperature rise, with the background subtracted (the heat adsorption of the pan and dry material). The integral was standardized by the weight loss during evaporation for each sample to calculate the unified evaporation heat, which was -17.36 J/g for PCN-641-NS and -6.14 J/g for PCN-642. The evaporation heat of PCN-641-NS is higher than that of PCN-642, which indicates the stronger adsorption of iodobenzene in the pores of PCN-641-NS.

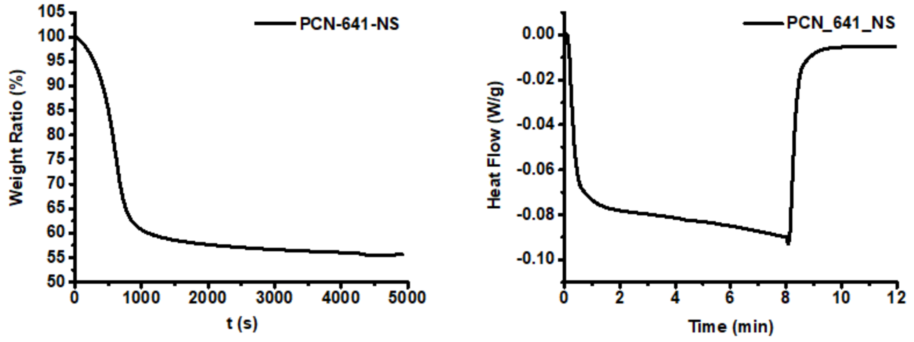

Figure S25. Weight variation (in weight ratio) of PCN-641-NS from room temperature to 80 °C.

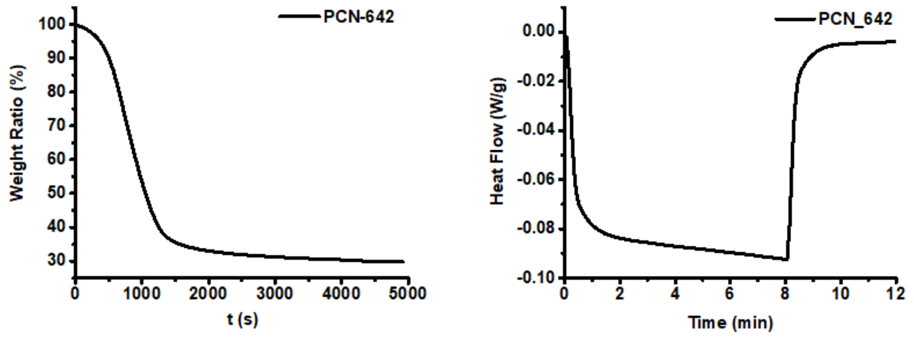

Figure S26. Weight variation (in weight ratio) of PCN-642 from room temperature to 80 °C.

Section 12. Photoreactions

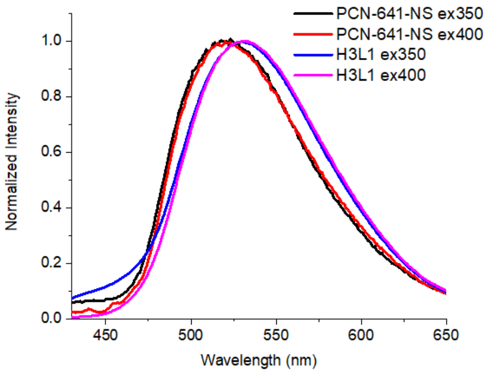

Figure S27. Photoluminescence of PCN-641-NS and H_3L1 ligand under 350 nm and 400 nm excitation.

All photoreactions were carried out in the light reaction container. The container is built with monochromatic light bands circling along the inner glass surface, as is shown in figure S24. The reaction mixture was charged into 20-mL scintillation vials with stirring bars which were then placed into the light reaction container.

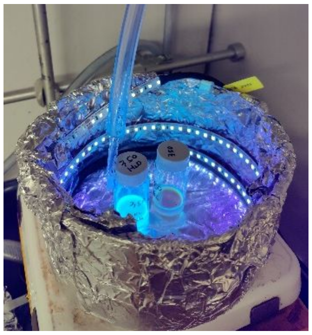

Figure S28. The photo-reactor setup. Monochromatic light band (350 nm) is taped on the inner surface of the glass container. The glass is fully wrapped with aluminum foil to block natural light. Air flow is connected to the reactor to cool the system and keep the reaction temperature at room temperature.

12.1 Photo-borylation

Iodobenzene (78.9 μmol), tributylamine (375 μmol), bis(pinacolato)diboron (375 μmol), 1,3,5-trimethoxybenzene (83.2 μmol) were mixed in acetonitrile (4.75 mL) and H_2O (0.25 mL) and charged into a 20-mL scintillation vial. PCN-641-NS (1.0 mg) was added into the mixture along with a stirring bar. The vials were kept stirring inside the photo-reactor. Test reactions were performed in 20 mL vials without gas exchange or water expulsion to demonstrate PCN-641-NS's tolerance for air and moisture. Iodobenzene, bis(pinacolato) diboron, tributylamine, and trimethoxybenzene (TMB, internal standard) were added in acetonitrile to PCN-641-NS for the experiments. In addition, water as a co-solvent and compared to the standard organic reaction test. For these tests a standard reaction system with 100 % MeCN was utilized alongside another system with 95% MeCN and 5% H_2O. Through high-performance liquid chromatography (HPLC), phenylboronic pinacol ester and phenylboronic acid were both detected in the reaction. It is worth noting that phenylboronic pinacol ester undergoes a gradual hydrolysis in the presence of H_2O towards

phenyl boronic acid. A total product yield (including phenylboronic pinacol ester and phenylboronic acid) of 86 % was observed in the 5% H_2O group, while the yield dropped to 49.0 % without H_2O added. This highlights the importance of water in the reaction transformation process. These findings indicate a possible reaction mechanism similar to PCC-40, which is a homogeneous porous coordination cage photocatalyst also developed in our group.[1] In comparison, PCN-641 and PCN-642 crystals were utilized in the same reaction conditions. Their slower reaction kinetics than PCN-641-NS in 5% H_2O acetonitrile indicate a mass transfer limit. The reaction conditions are summarized in Table S3.

Table S3. Reaction conditions studied in photo-borylation.

Batch number	Catalyst	Excitation light	Solvent
1	None	350 nm	5% H_2O acetonitrile
2	PCN-641-NS	350 nm	5% H_2O acetonitrile
3	PCN-641-NS	350 nm	acetonitrile
4	PCN-641	350 nm	5% H_2O acetonitrile
5	PCN-642	350 nm	5% H_2O acetonitrile

The PXRD pattern of PCN-641-NS pre- and post-reaction showed a maintenance of the (100) peak, which is the major peak for the 2D framework, demonstrating its stability. The slight shift in other peaks indicates slight crystallinity alterations during the catalysis. The reaction products were monitored by HPLC.

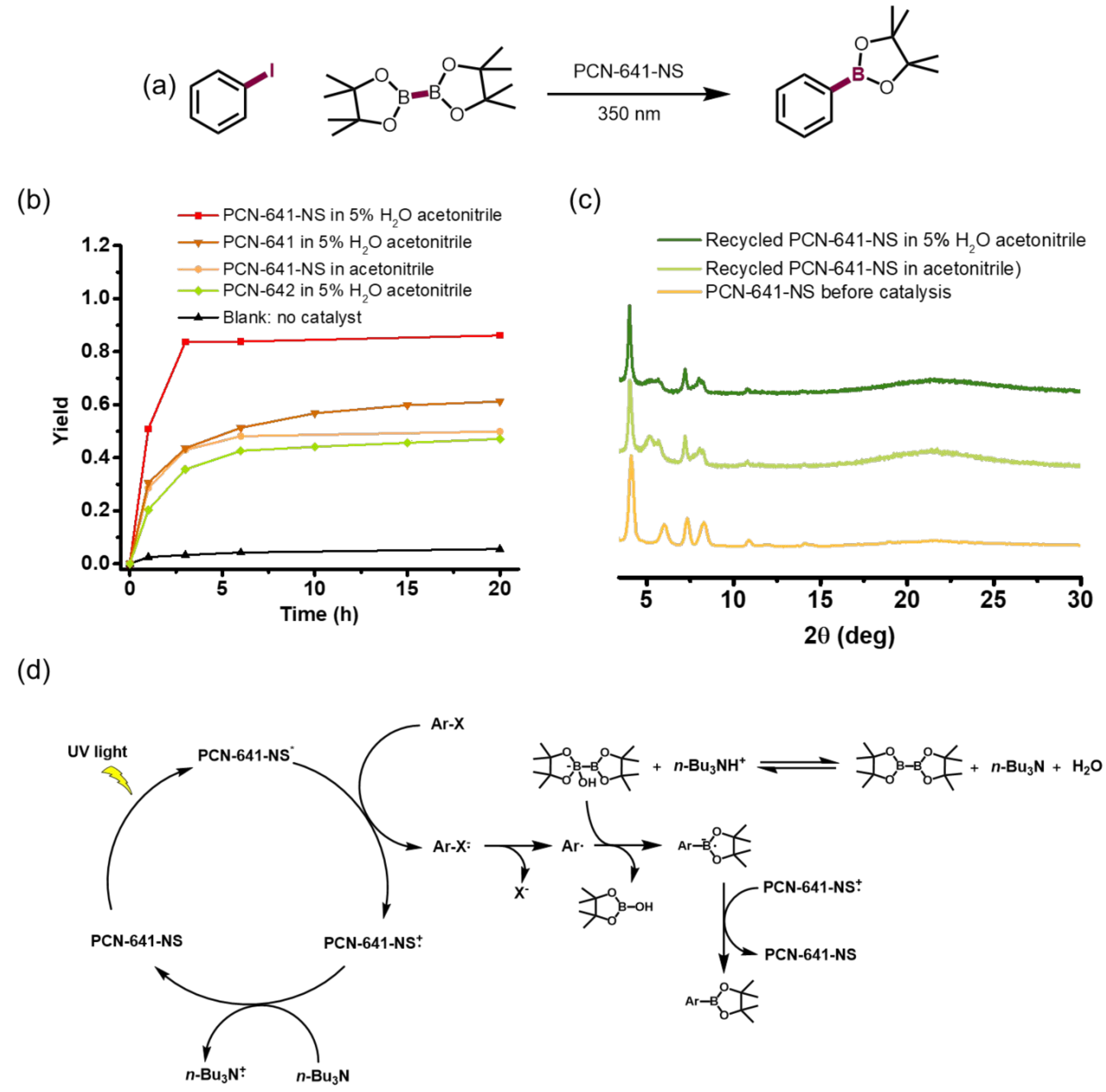

Figure S29. HPLC monitored photo-borylation of iodobenzene catalyzed by photocatalysts. (a) Reaction scheme for photo-borylation (b) Photo-borylation kinetics under different conditions. (c) PXRD patterns of PCN-641-NS before and after catalysis. (d) Proposed reaction mechanism based on single-electron transfer.

12.2 Photo-induced $^1O_2$ generation

Diphenylisobenzofuran (DPBF) was dissolved into THF to obtain a solution of 10 mg/mL. PCN-641, PCN-642, PCN-641-NS, PCN-643-NS, and PCN-644-NS were added to THF to yield 1 mg/mL solutions, separately. They were later diluted 5 times to yield 0.2 mg/mL solutions in 2 mL 10 mg/mL DPBF solutions. The solution was transferred to quartz fluorescence cuvette and placed in photo reactor (λ_{ex} = 350 nm) shown in Figure S24. The reaction time was recorded, and the fluorescence spectra were collected on a Horiba Fluorescence Spectrophotometer with λ_{ex} = 410 nm (DPBF's excitation wavelength). The reaction conditions are summarized in Table S4. The fluorescence spectra and fitting curves are summarized in Figure S26.

Table S4. Reaction conditions studied in photo-borylation.

Batch number	Catalyst	Excitation light	Solvent
1	None	350 nm	Degassed THF
2	PCN-641		THF
3	PCN-642		THF
4	PCN-641-NS		THF
5	PCN-643-NS		THF
6	PCN-644-NS		THF

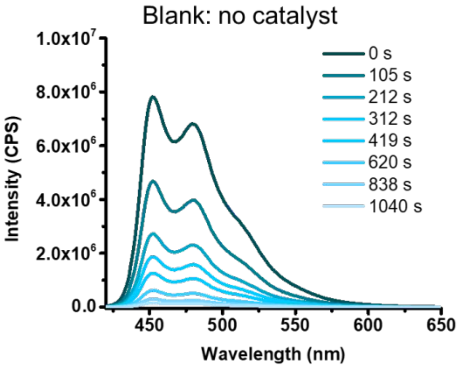

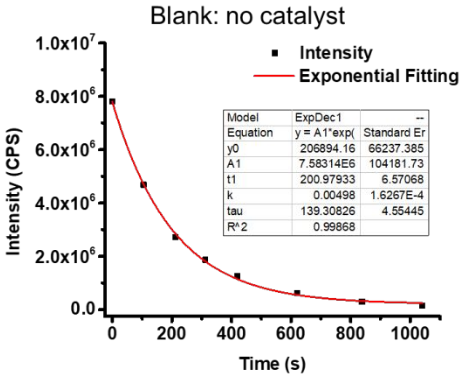

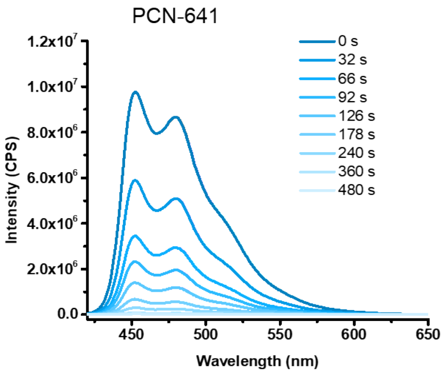

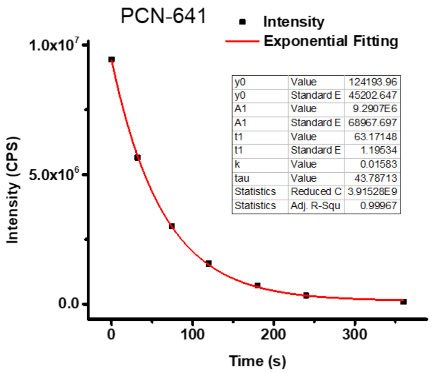

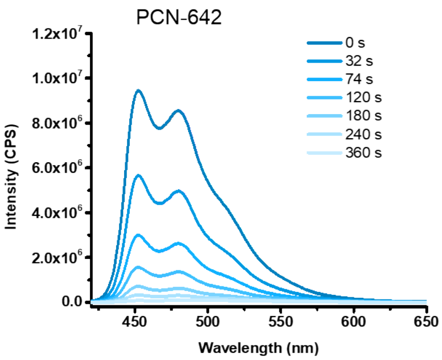

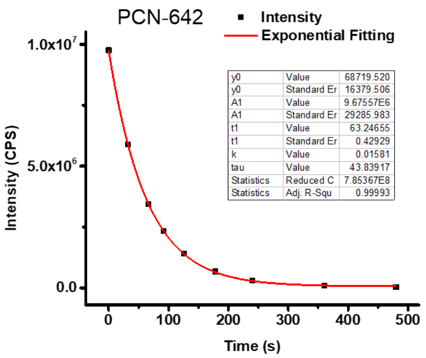

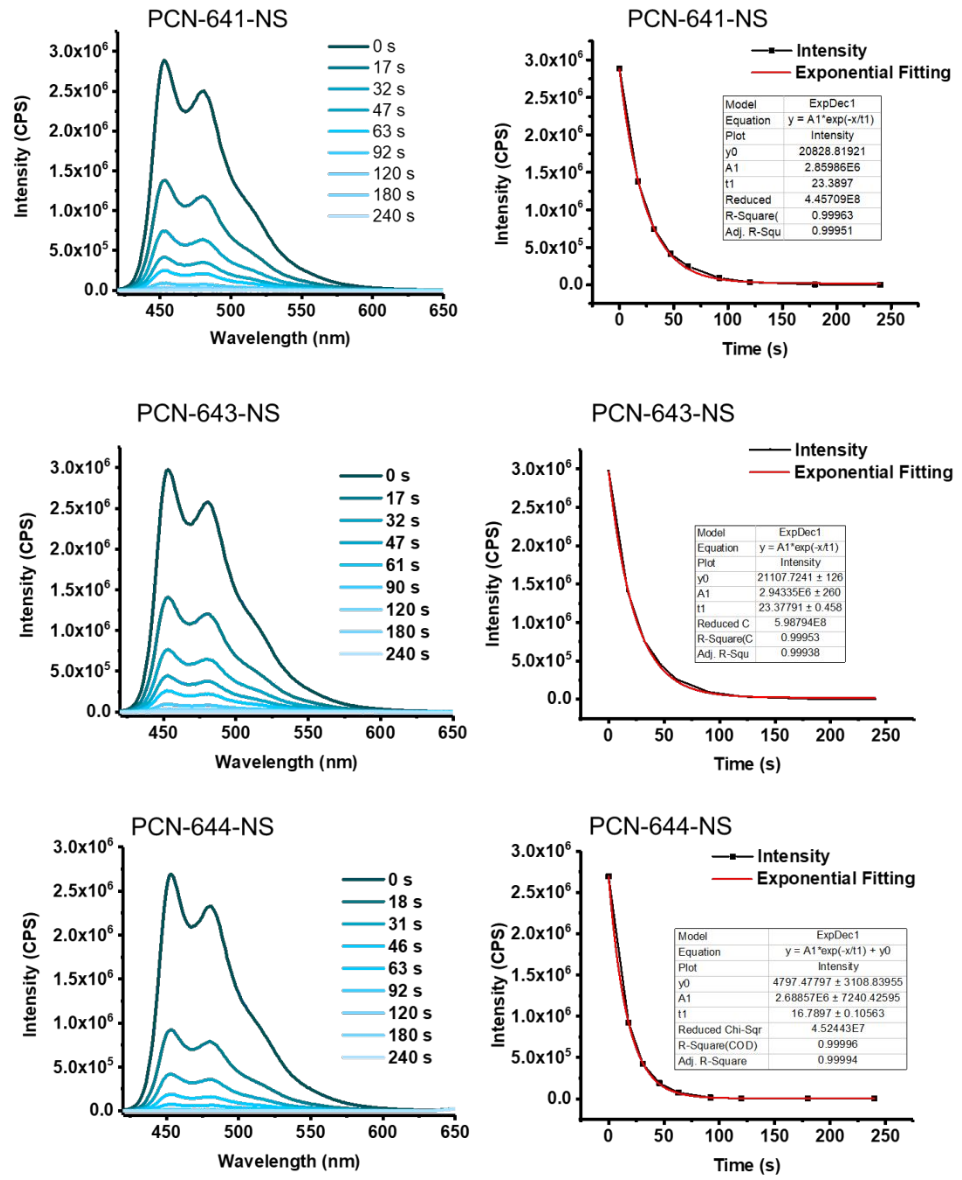

Figure S30. DPBF fluorescence quenching monitored reaction kinetics of $^1O_2$ generation. Exponential fitting was adopted, and the yielded parameters summarized in each figure.

Section 13. Electric Conductivities

The electric conductivities of PCN-641-NS and PCN-641-NSO were measured by Keithley 4200 using the 4-point probe method. I_2 doping of the material was conducted by placing the nanosheet materials in a 4-mL vial with I_2 solid in a larger 50 mL sealed container and heated at 40 °C for 2 hours. An increase in electric conductivity was observed. The pellet thickness was collected by a KLA-Tencor P-6 Stylus Profilometer (JCAIN 308) and are labelled in Figure S27.

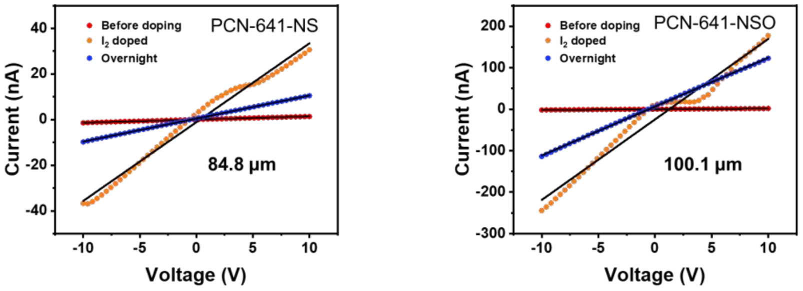

	I/V (nA/V)	Conductivity (S/cm)
**Before**	0.138	3.61E-09
**Doped**	3.46	9.00E-08
**Overnight**	1.01	2.63E-08

	I/V (nA/V)	Conductivity (S/cm)
**Before**	0.175	3.86E-09
**Doped**	19.4	4.28E-07
**Overnight**	11.8	2.60E-07

Figure S31. Electric conductivities measured by a 4-point probe. The conductivities are measured before I_2 doping, with I_2 doping, and overnight after doping.

Section 14. Band Structure Simulation

All periodic calculations were performed within the Kohn-Sham DFT framework using the Vienna ab initio Simulation Package (VASP 5.4.4) with a plane wave cut off of 500 eV at the gamma point. The structures were geometrically optimized using the PBEsol functional.[6] The structures with increasing interlayer spacing were optimized with respect to their ionic positions and unit cell shape, while keeping cell volume constant. The ionic and electronic convergence criteria were 0.005 eV and 10^{-5} eV, respectively. Density of states were calculated from the optimized structures using HSEsol functional with the same basis.[7] Band structures were calculated from the optimized structures using PBEsol.

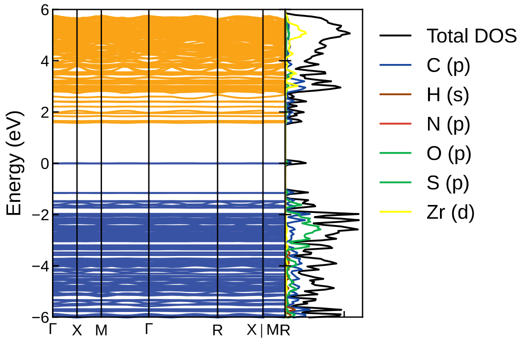

Figure S32. PBEsol band diagram and density of states of AA stacked material.

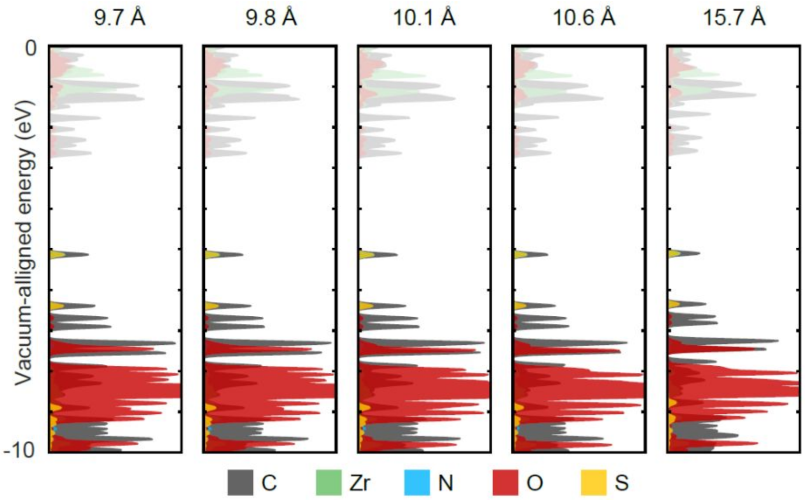

Figure S33. Density of states of AA stacked material at different inter-layer distances.

1. Lin, H.; Xiao, Z.; Le, K. N.; Yan, T.-h.; Cai, P.; Yang, Y.; Day, G. S.; Drake, H. F.; Xie, H.; Bose, R.; Ryan, C. A.; Hendon, C. H.; Zhou, H.-C., Assembling Phenothiazine into a Porous Coordination Cage to Improve Its Photocatalytic Efficiency for Organic Transformations. *Angew. Chem. Int. Ed.* **2022,** *61* (49), e202214055.
2. Feng, D.; Gu, Z.-Y.; Li, J.-R.; Jiang, H.-L.; Wei, Z.; Zhou, H.-C., Zirconium-Metalloporphyrin PCN-222: Mesoporous Metal–Organic Frameworks with Ultrahigh Stability as Biomimetic Catalysts. *Angew. Chem. Int. Ed.* **2012,** *51* (41), 10307-10310.
3. Wang, T. C.; Vermeulen, N. A.; Kim, I. S.; Martinson, A. B. F.; Stoddart, J. F.; Hupp, J. T.; Farha, O. K., Scalable synthesis and post-modification of a mesoporous metal-organic framework called NU-1000. *Nature Protocols* **2016,** *11* (1), 149-162.
4. Sheldrick, G., A short history of SHELX. *Acta Crystallographica Section A* **2008,** *64* (1), 112-122.
5. Spek, A., Single-crystal structure validation with the program PLATON. *J. Appl. Crystallogr.* **2003,** *36* (1), 7-13.
6. Perdew, J. P.; Ruzsinszky, A.; Csonka, G. I.; Vydrov, O. A.; Scuseria, G. E.; Constantin, L. A.; Zhou, X.; Burke, K., Restoring the Density-Gradient Expansion for Exchange in Solids and Surfaces. *Phys. Rev. Lett.* **2008,** *100* (13), 136406.
7. Schimka, L.; Harl, J.; Kresse, G., Improved hybrid functional for solids: The HSEsol functional. *The Journal of Chemical Physics* **2011,** *134* (2).
